# BMC ecology image competition 2017: the winning images

**DOI:** 10.1186/s12898-017-0138-8

**Published:** 2017-08-18

**Authors:** Christopher Foote, Chris T. Darimont, Michel Baguette, Simon Blanchet, Luke M. Jacobus, Dominique Mazzi, Josef Settele

**Affiliations:** 1 BMC Ecology, BioMedCentral, London, UK; 2 Department of Geography, University of Victoria, Victoria, Canada; 3 Institut de Systématique, Evolution et Biodiversité, Muséum National d’Histoire Naturelle (MNHN), UMR 7205, Paris, France; 4 Station d’Ecologie Théorique et Expérimentale du CNRS, Centre National pour la Recherche Scientifique (CNRS) & Université Paul Sabatier (UPS), Toulouse, France; 5 Division of Science, Indiana University Purdue University Columbus (IUPUC), Columbus, USA; 6 Federal Department of Economic Affairs, Education and Research EAER, Agroscope, Research Division Plant Protection, Nyon, Switzerland; 7 UFZ Centre for Environmental Research, Leipzig, Germany; 8 German Centre for Integrative Biodiversity Research (iDiv), Halle-Jena-Leipzig, Leipzig, Germany; 9 Institute of Biological Sciences, University of the Philippines Los Baños, College, Laguna, 4031 Philippines

## Abstract

For the fifth year, *BMC Ecology* is proud to present the winning images from our annual image competition. The 2017 edition received entries by talented shutterbug-ecologists from across the world, showcasing research that is increasing our understanding of ecosystems worldwide and the beauty and diversity of life on our planet. In this editorial we showcase the winning images, as chosen by our Editorial Board and guest judge Chris Darimont, as well as our selection of highly commended images. Enjoy!

**Electronic supplementary material:**

The online version of this article (doi:10.1186/s12898-017-0138-8) contains supplementary material, which is available to authorized users.

## Editorial

The 2017 edition of our image competition has produced a terrific array of images of the natural world, from close-ups that capture the animated life of insects to aerial views of vast landscapes. As with previous editions of the competition [1–4] we are thrilled at the diversity and excellence of the submissions we received from ecologists across the world.

This year we are delighted to have as our guest judge Chris Darimont of the University of Victoria, Canada. As well as producing high quality interdisciplinary applied research for the benefit of natural and human systems, Chris has demonstrated an admirable commitment to outreach and science communication. This, combined with his considerable enthusiasm for photography, made him an excellent judge to help select the very best from the submissions we received this year.

Alongside Chris, once again our *BMC Ecology* Section Editors lent their expertise to our competition, picking out their favourite images in their areas of speciality. Having the input of such respected scientists as our judges ensures our winning images are picked as much for the scientific story behind them as for the technical quality and beauty of the images themselves.

## Winning images

**Fig. 1:**
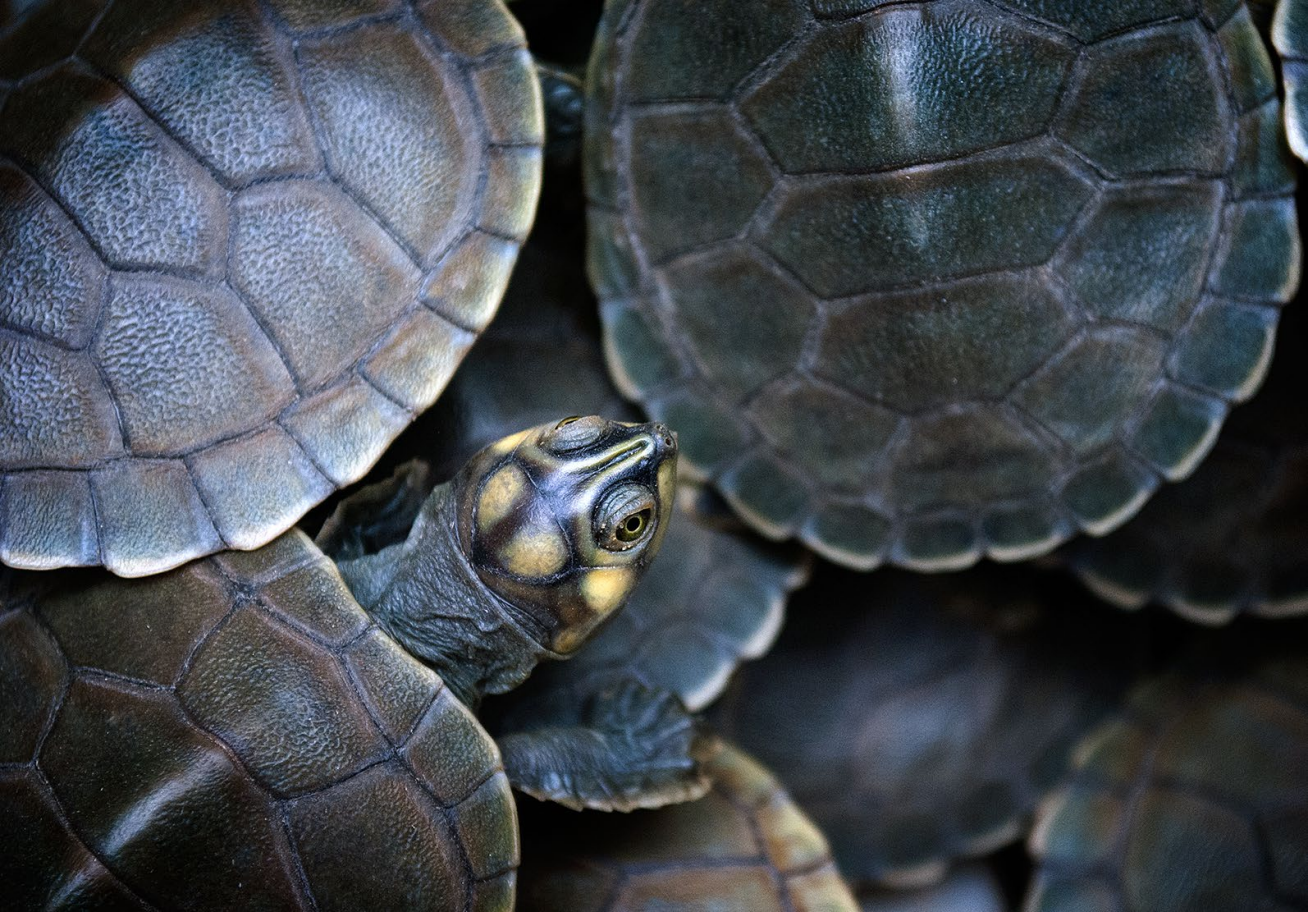
Overall winner: *Podocnemis expansa*. *“The Giant South American turtle (Podocnemis expansa), also known as the tartaruga*-*da*-*amazônia, is the largest of the side*-*neck turtles. This picture was taken in one of the most important areas of conservation for this species: the Cantão State Park, in Tocantins, Brazil. The Park is located in the Brazilian “Savanna” or Cerrado, a biodiversity hotspot that is yet poorly known but is thought to be equally or even more biodiverse than the Amazon ecosystem. I was there as part of a research group working in the field to collect data on the status of reptiles and amphibians’ populations for conservation purposes.”* Attribution: Ana Carolina Lima

Our overall winning image this year was Ana Carolina Lima’s photo of giant South American turtles (*Podocnemis expansa*). Chris Darimont admired the “…rare, multi-layered perspective from above. The photo is well composed, technically sound, and rich with wonderful geometry” (Fig. 1).

Our Conservation Ecology and Biodiversity Section Editors Luke Jacobus and Josef Settele also admired this image, not just for the photo itself, but also for the story behind it. Dr. Lima, based at the University of Aveiro, Portugal, conducts her research in the Brazilian Cerrardo. This region is the largest savanna in South America and has been recognized as one of the 25 biodiversity hotspots of the world [5, 6]. It holds considerable (and still considerably unquantified) biodiversity, yet is increasingly threatened by human development. As Luke Jacobus said of the image, “its story best communicates the ideas of biodiversity and conservation. Brazil, in particular, is facing certain challenges to conservation of its biodiversity, and we stand to learn much about the Cerrado so that loss of its diversity can be minimized.”

This photo, alongside another image by Dr. Lima of a frog from the family Leptodactylidae (included among our highly commended images), does a fantastic job of showcasing the diversity of the Brazilian Cerrardo that her research contributes to conserving. It is a worthy winner of this year’s competition.

## Runners up

**Fig. 2:**
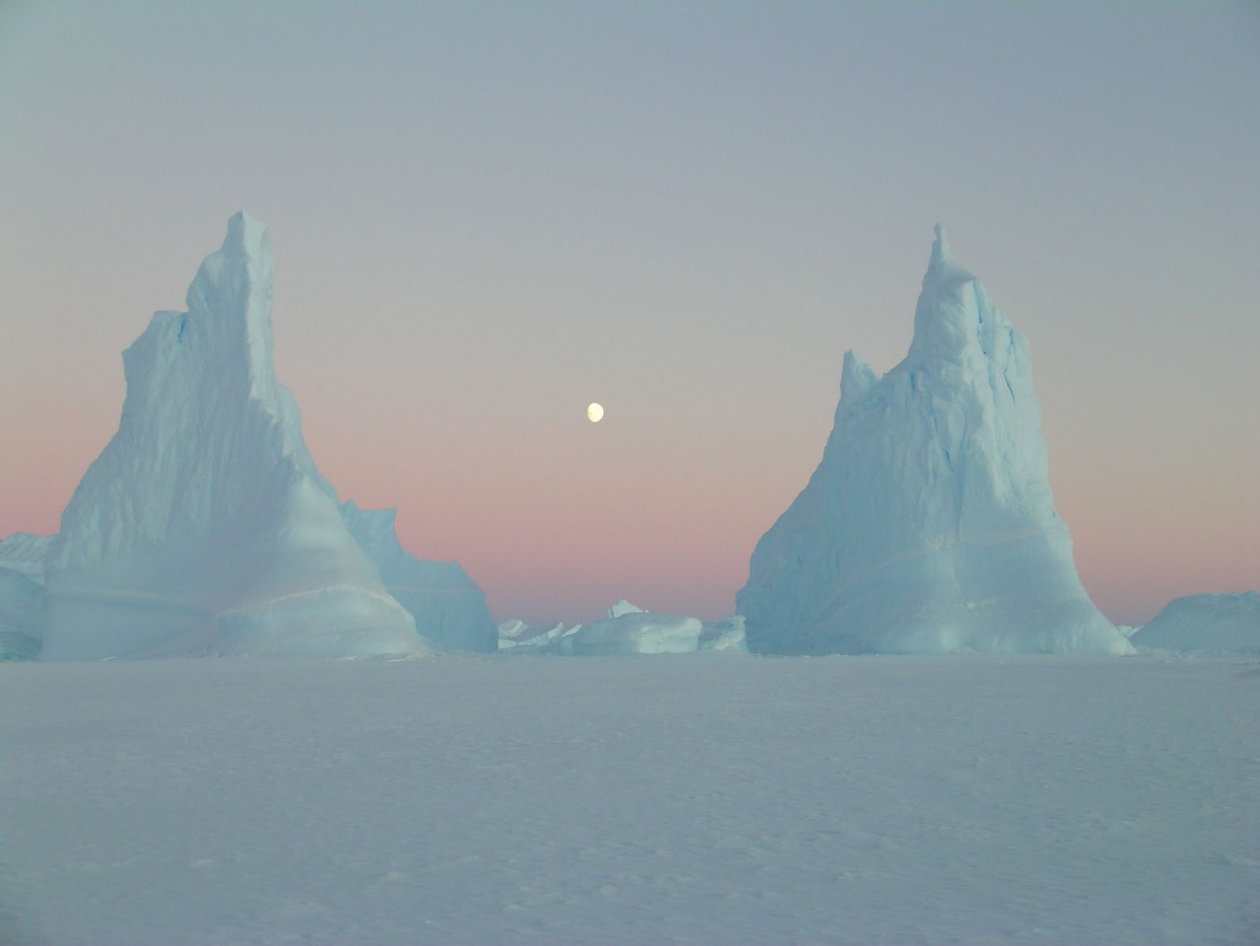
First runner-up: Two towers. *“The tranquil and frozen East Antarctic sea ice landscape in the winter months showing off its amazing pink skies and fantastic icebergs.* *During my PhD I spent over a year at Davis station in the Australian Antarctic Territory doing research on two freshwater lakes in the Antarctic lake oasis known as the Vestfold Hills. I was lucky to capture these “two towers” and the striking moon on a sea ice trip near the Davis station in June 2004.”* Attribution: Christin Säwström

Our first runner-up presents quite a contrast to our overall winner. Whereas the image of turtles presented a snapshot of life in tropical rainforests, teeming with life, Christin Säwström’s (Edith Cowan University, Western Australia) photo ‘Two towers’ captures the austere landscape of the Antarctic (Fig. 2).

Chris Darimont commented that the image is “not sharp in the low light environment, an effect that gives it a winter dreamy feel. Also, because of its softer edges, it resembles a painting. Owing to this uncertainty, it invites close and sustained examination. Whether by design or happenstance, this is a rare approach that produced an arresting image.”

**Fig. 3:**
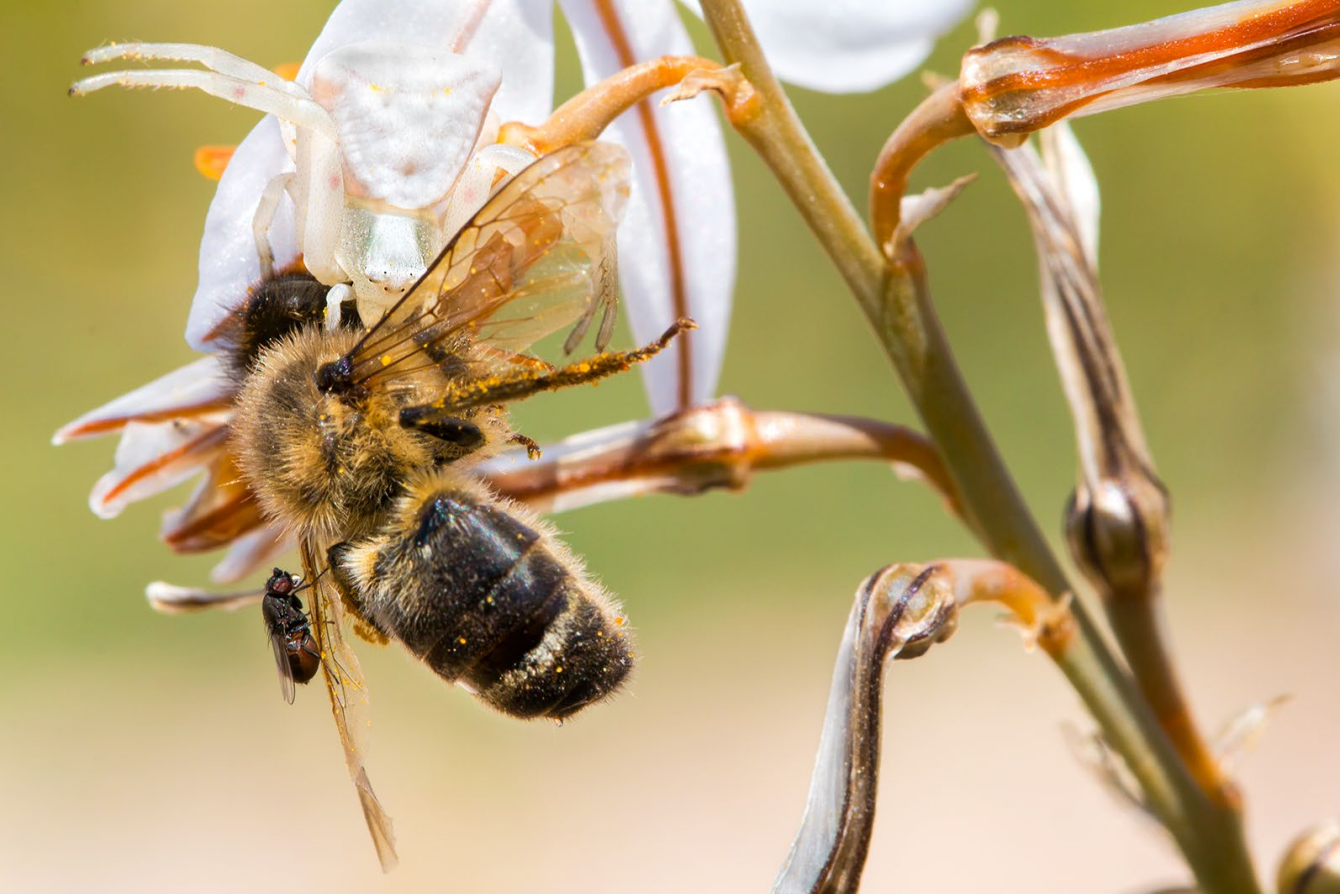
Second runner-up: Connections. *“Conservation cannot be understood without taking into account the interaction among species. The* *disappearance of one species might provoke that other connected species (prey, predators,* etc*.) can suffer direct consequences. This picture represents the predation of a crab spider on a bee and this was used by a parasitic fly.”* Attribution: Roberto García-Roa

Our second runner-up, an image by Roberto García-Roa of the University of Valencia, Spain entitled ‘Connections’, shows a remarkable series of ecological interactions (Fig. 3). A predatory spider, camouflaged by the white plant on which it hunts, has caught a large bee, which is also being attacked by a parasitic fly. As Chris Darimont put it, “its title sums up what I like best here. Typically in pictures with animals, one is drawn to the eyes of the larger, charismatic subjects, in this case the spider or bee. In this image, however, the star is the smaller fly. This parasite, tack sharp, commands the attention it deserves as a major player in this interaction and in ecosystems in general.”

Our Section Editor Simon Blanchet also enthused about this photo: “This picture, by encapsulating a four-way species interaction, perfectly illustrates the complexity of species interactions by illustrating the dependence of species on each other, but also the fabulous power of evolution to optimize all the energy available in a food web and to generate aesthetic entities.”

## Behavioral ecology and physiology

**Fig. 4:**
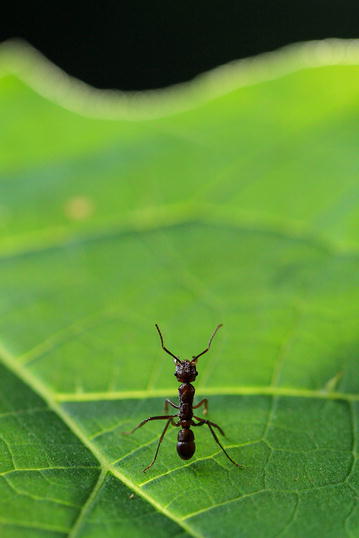
Winner, Behavioral and Physiological Ecology: Wakeful. *“Photograph taken in forest in French Guiana. An ant (Ectatomma sp) on alert defending its territory on a leaf.”* Attribution: Maïlis Huguin

Our winner in this category was Maïlis Huguin from the Institut Pasteur de la Guyane, French Guiana with his image, ‘Wakeful’, of a lone ant defending its territory (Fig. 4).

Section Editor Dominique Mazzi admired “an appealingly simple image with the two colors alternating in strong contrast and a succeeded crossing from sharp to un-sharp. The ant stands at attention with a determination that suggests that the leaf it is watching over is the most important thing in the whole world. At the same time, the ant calls for the viewer’s attention as if claiming that if it was not for its guarding it, then the most important thing in the whole world would be lost.”

Chris Darimont also liked the impression the ant gives us: “It appears territorial but also says to me, ‘I’m an individual and I count’, which protests against the reality that this individual is very likely a tiny and subjugated part of a larger social whole.“

## Community, population and macroecology

**Fig. 5:**
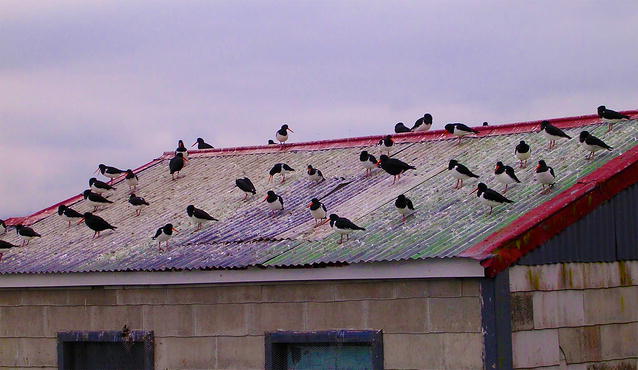
Winner, Community, Population and Macroecology: Catchers on a hot tin roof. *“Early morning oyster catchers assemble on the roof of a disused boat shed on the Otago Peninsula, South Island, New Zealand”* Attribution: Trevor Sherwin

“L’avenir appartient à ce qui se lèvent tôt”—the future belongs to those who get up early. This French saying was brought to mind for our Section Editor Simon Blanchet, as he chose the winning image for the Community, Population and Macroecology category, entitled ‘Catchers on a hot tin roof’ (Fig. 5).

The photo of a group of oyster catchers assembling in the morning sun, taken by Professor Trevor Sherwin of the University of Auckland, New Zealand, was particularly liked by Simon for the “colors of this early morning picture. At the least it is for those unique colors and pictures that we (sometimes) get up early.”

## Conservation ecology and biodiversity

**Fig. 6:**
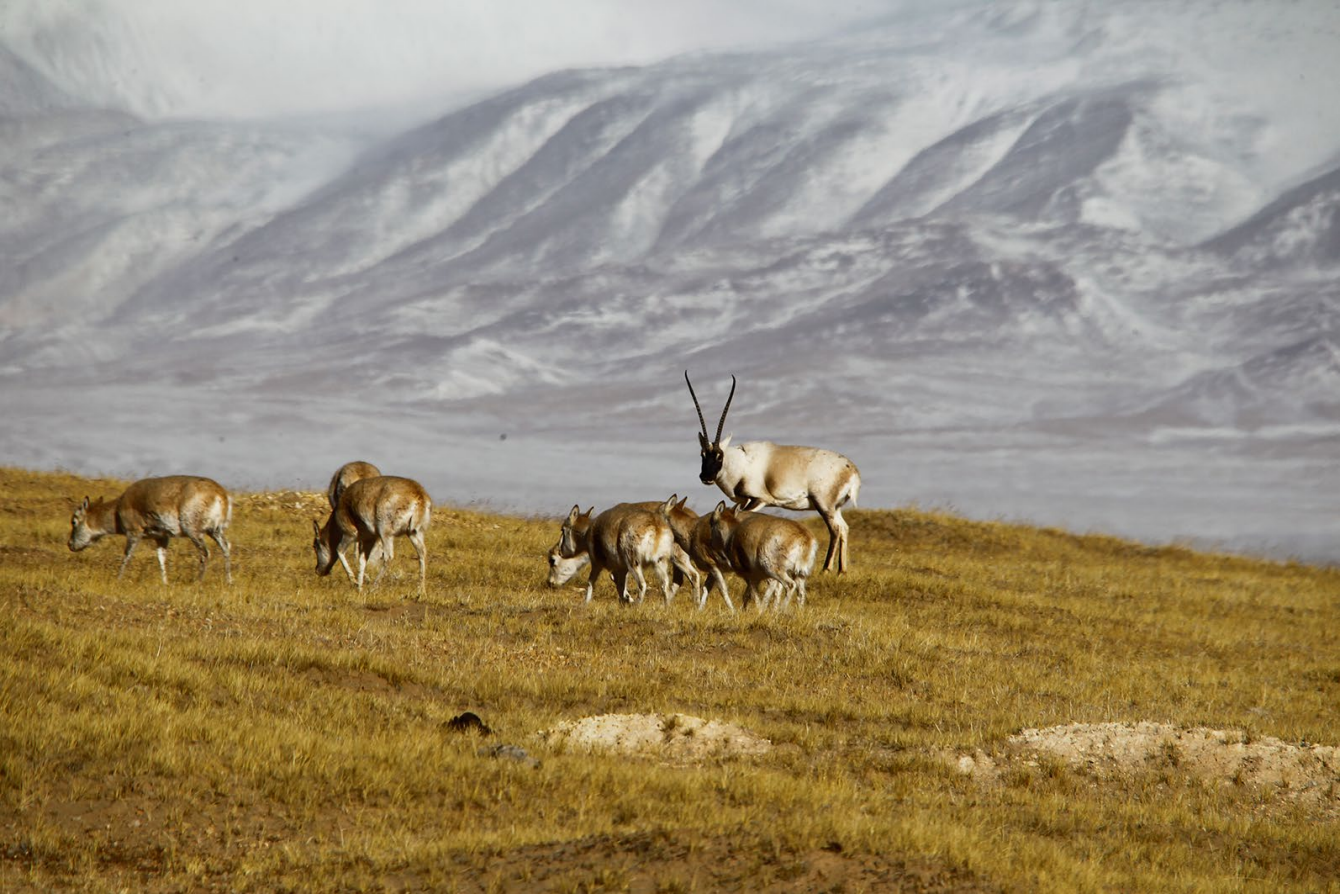
Winner, Conservation ecology and biodiversity: Tibetan antelope. *“On January 12, 2016, at the foot of white snow covered Kekexili Mountain, a male Tibetan antelope was vigorously guiding a herd of female which would come to estrus soon. Tibetan antelope have a harem mating system, in which a dominant male who defeated other male competitors during rut will have a harem of several to dozens of females during the rutting season in the coldest month of the year. Tibetan antelope are an endemic species to the Qinghai*-*Tibetan Plateau. Its population once reached several millions on the alpine meadows in the heartland of the plateau. However, the population of Tibetan antelope dramatic decreased to 70*–*80 thousands after the mad poaching for its precious wools at the end of 20th century. Since then, populations of Tibetan antelope (Chiru, Pantholops hodgsonii) have been gradually recovering under strict protection. The status of Tibetan antelope was down listed from “Critically endangered” to “Valuable” in the 2015 China’s Biodiversity Red List.”* Attribution: Zhigang Jiang

Chosen by Section Editors Luke Jacobus and Josef Settele, our winner in this category was Professor Zhigang Jiang of the Institute of Zoology, Chinese Academy of Sciences, China with his image of a male Tibetan antelope guarding his harem of females (Fig. 6).

Josef Settele commented that this is “a species and ecosystem that is often underrepresented” and is a reminder of the importance of Chinese conservation efforts in this fragile ecosystem. We also admired the composition of the photo, with the females facing away from us, but the male glaring directly at the camera as though preparing to defend his harem.

## Landscape ecology and ecosystems

**Fig. 7:**
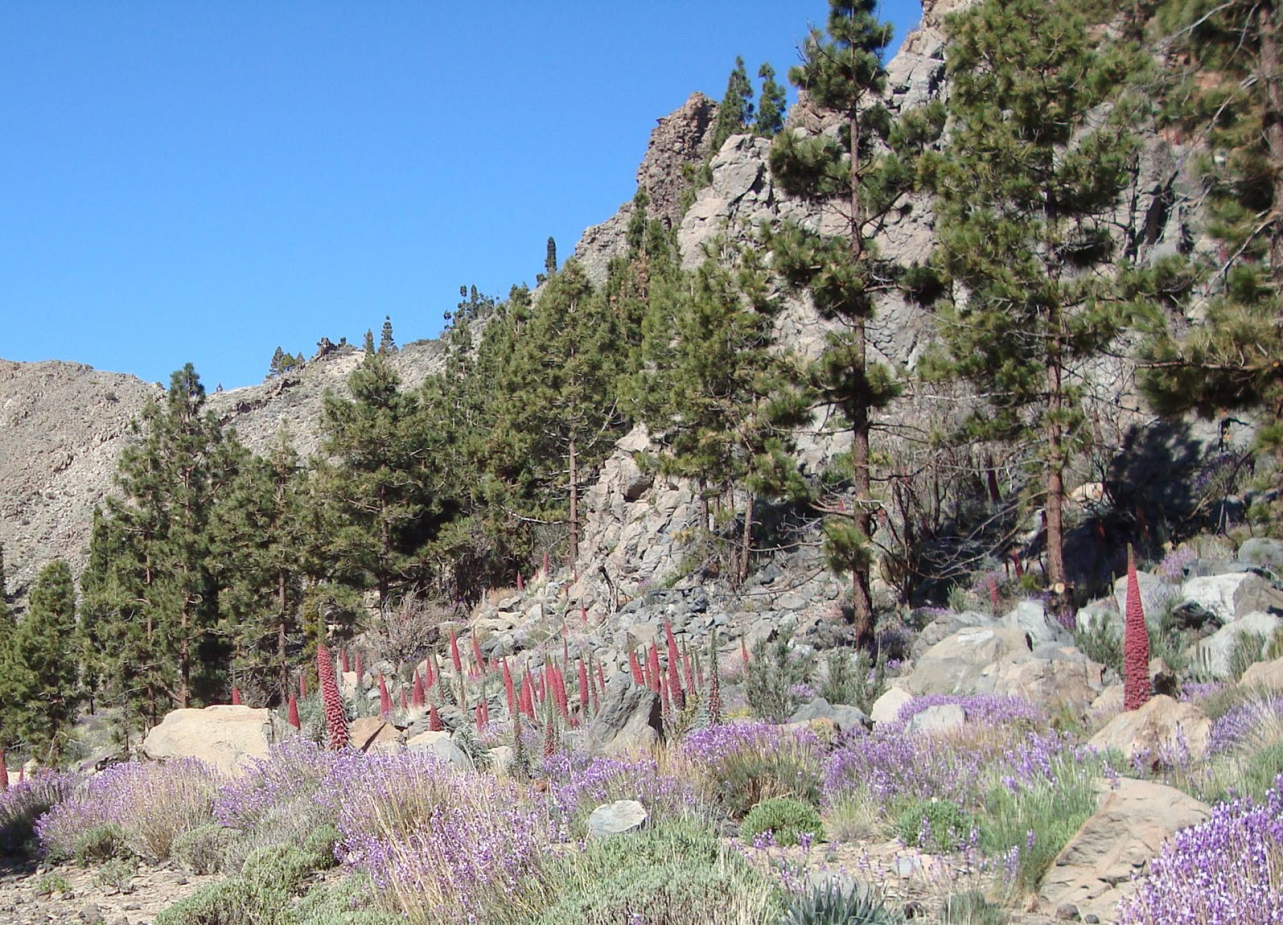
Winner, Landscape ecology and ecosystems: Las Cañadas del Teide. *“Micro*-*habitat on the north*-*facing caldera slopes at 2100* *m altitude in ‘Las Cañadas del Teide’ on Tenerife Island, Spain. Shown here is a rare mixture of the Canary pine (Pinus canariensis) and the flowering endemic Teide bugloss (Echium wildpretii), amongst other plants, such as the rosallilo de cumbre (Pterocephalus lasiospermus). The unique geological environment and geomorphological development created a specific micro*-*habitat, perfectly suitable for both the Canary pine trees invading from outside the caldera depression and the ‘Tower of Jewels’ or ‘Red bugloss’, a rare species fully adapted to survive in this harsh terrain. The micro*-*habitat is formed by coarse rock fall deposits, piling up at the foot of the* >*3 million years old basaltic cliffs, creating local scree slopes. Locally, fine*-*grained sub*-*horizontal soil patches form behind larger fallen blocks, as the result of fine*-*scale surface runoff and sediment transport and deposition, which, in combination with mechanical and chemical weathering, release sufficient nutrients from the basaltic parent material to sustain this unique vegetation cover. Signs of forest fire, especially on the pine trees, emphasize the vulnerability and dynamic nature of this ecologically fragile (micro*-*)ecosystem.”* Attribution: Harry Seijmonsbergen

The winning image in this category was an image from Mount Teide, the huge volcano located on Tenerife, captured by Professor Harry Seijmonsbergen from the University of Amsterdam, Holland (Fig. 7).

Our Section Editor Michel Baguette explained his choice: “The Canary islands are located 100 km off Morocco and have a total area of 7500 km^2^. Of the 1700 plant species recorded on the seven volcanic islands of the archipelago, more than 500 are endemic to this small area. There are also endemic animal species, including reptiles, birds and mammals. The beauty of many endemic species, like the Teide bugloss (*Echium wildpretii*) or the blue chaffinch (*Fringilla teydea*) should not make us forget how fragile they are. Endemic species have usually a low number of individuals and are restricted to small areas that are threatened by human activities. This is perfectly exemplified by the description written by Harry Seijmonsbergen of the complex processes required for the creation of the microhabitat illustrated in the winning picture. The preservation of endemic species on islands is thus a paradigm for biodiversity conservation everywhere on Earth.”

## Editor’s pick

**Fig. 8:**
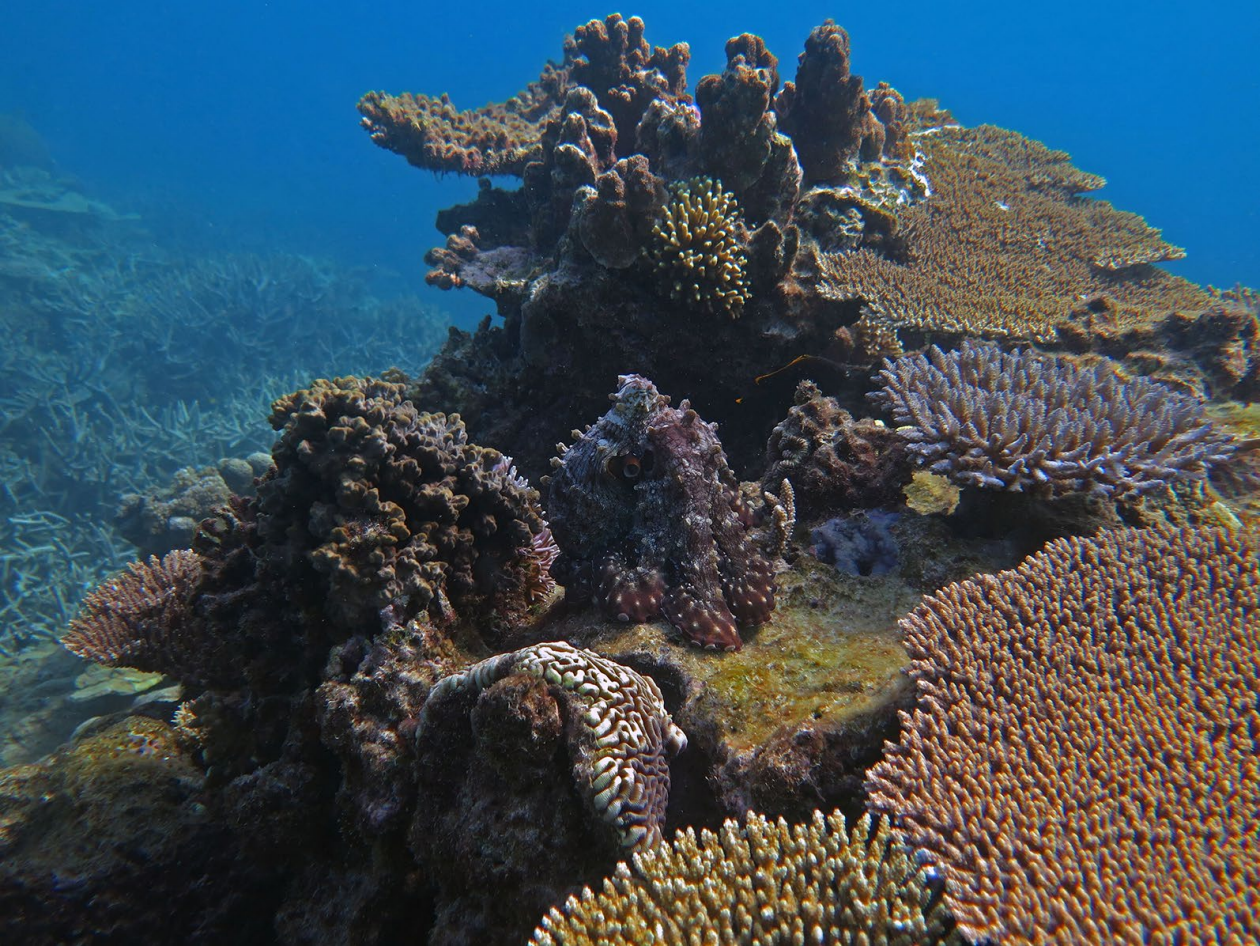
Winner, Editor’s pick: A “well-armed” coral reef community. *“The underwater equivalent of tropical rainforests, healthy coral reefs teem with life. Often conspicuous, other times a little harder to spot. Like this octopus, that pretended to be a coral as I approached it. The complex three*-*dimensional framework that corals build provides a sheltered habitat and ideal camouflage ground to approximately ¼ of all marine species, even though reefs are just specks in our oceans. It is hard to imagine however how a bleached or dead reef could host such biodiversity, an alarming thought given the back*-*to*-*back global bleaching events of 2016 and 2017. Heron Reef, along with other reefs in the southernmost section of the Great Barrier Reef, may have escaped the bleaching that hit the warmer northern section this time round, but how well*-*armed these reefs are against intensifying climate change and other anthropogenic pressures is questionable.“*Attribution: Michelle Achlatis

If ecology is the art of uncovering connections within ecosystems that are not immediately obvious, then our Editor’s pick, entitled ‘A “well-armed” coral reef community’, represents this perfectly (Fig. 8). At first the image, by Michelle Achlatis of the University of Queensland in Australia, seems to present a coral reef surprisingly lacking in animal life; until, that is, you notice the large octopus hidden in plain sight (*if you are having trouble finding it, look for the eye exactly in the middle of the picture*).

Aside from the enjoyment of playing ‘spot the octopus’, the picture is also a reminder of how integrated the different species are within coral ecosystems; this octopus, so beautifully adapted to its environment, could clearly not survive outside this habitat. With coral reefs increasingly under threat, let’s hope in years to come the difficulty in spotting such octopi will still be due to their expert camouflage and not because they have become extinct.

## Highly commended

Beyond our winning images we received many other fantastic photos. We highlight our favourites in this section.

The first is by Pablo Juarbe Martinez, a photo of a Galapagos sea lion dozing in the sand—simple, yet striking (Additional file 1). José López Bucio contributed a colorful image of flowering epiphytic plants in his ‘candle in the wind’ (Additional file 2). Initially, ‘Dicing with death’ by James O’Hanlon also looks like another photo of a colorful plant. Closer inspection reveals a well-disguised Malaysian orchid mantis—and a fly lucky to have avoided becoming its meal (Additional file 3).

A rather larger scale vision of nature was provided by Diogo Sayanda’s aerial view of the Sado River estuary in southern Portugal (Additional file 4). Chris Darimont admired the “gorgeous color and composition. One need’s to know it is an estuary from the title to know it is an aerial shot (and that’s a good thing). It invites closer inspection, which take you to the ‘oh-ya!’”.

## Life and death

A number of submissions documented the effort species undergo to pass their genes down to the next generation. Michaël Nicolaï contributed a lovely image of a male frog perched on a stem, awaiting any females responding to his call (Additional file 5). Arnaud Badiane photographed two mating common wall lizards; Chris Darimont noted the “intense look from one of the lizards, presumably the male, with his limb thrown around the female” (Additional file 6).

A slightly different male–female relationship was captured by Maïlis Huguin in his image of a male and female spider entitled ‘Sexual Dimorphism’ (Additional file 7). As Chris Darimont put it: “Sexual Dimorphism—clear case of exactly that!”. Demonstrating that mating needn’t be all consuming, Jeroen Everaars photographed a female dancefly feeding on its prey while simultaneously mating with a male—who, seemingly precariously, keeps the three of them from falling to the ground (Additional file 8).

For the swallow-tailed gull, mating involves a substantial lifestyle change. The gulls spend most of their lives out at sea, only returning to land to breed. Majoi de Novaes Nascimento captured a gull perched high up on a cliff face at their breeding grounds on the Galapagos Islands (Additional file 9).

For many species, mating is the easy part; taking care of the resulting offspring is where it gets tricky. Roberto García-Roa captured this reality brilliantly with his photo of the frog *Oophaga pumilio* carrying its tadpole on its back, searching for a safe pool in which to deposit it (Additional file 10). The composition makes us appreciate the immensity of the rainforest compared to the tiny frog.

Producing young is normally the only job for ant queens, but as Julia Giehr’s colorful photo demonstrates, when they need to they will pitch into save their offspring (Additional file 11). Diogo Sayanda’s photo shows a male *Abudefduf luridus* trying—and failing—to save his eggs from a shoal of predatory fish (Additional file 12). Perhaps it’s wrong for us to find such an image so beautiful, but as Chris Darimont said “Oh—those gorgeous colors”!

For some species, reproduction and predation are one and the same, as with *Cordyceps* fungi, which parasitize insects in order to reproduce. The image ‘Fungus Attack’ (a third contribution by the very talented Maïlis Huguin) shows a butterfly parasitized in this way (Additional file 13). Chris Darimont felt the picture “captured the beauty (especially the colors) in an otherwise macabre process of decay. I also liked the apparent suddenness with which the moth expired, its feet seemingly glued by fungus to the leaf”.

## The human factor

Several images highlighted how humans can affect natural processes—or vice versa, as in the case of a photo by Mohd Masri bin Saranum of a *Cryptolaemus montrouzieri* larva eating mealybug larvae (Additional file 14). While the *Cryptolaemus* larvae may look rather monstrous, it is actually an effective natural control against mealybugs, a serious pest of papaya plants. Simon Blanchet commented that this image “illustrates the complexity of life and the strength of evolution in shaping species interactions and food webs”.

Our judge Josef Settele meanwhile liked “the industry-related story plus the marvellous picture” of Rozilawati binti Harun’s image of a stingless bee (Additional file 15).

Hannah Bose contributed a lovely image of periwinkles making their home alongside humans on a busy beach in the Seychelles (Additional file 16). Chris Darimont liked the “nice composition of wood arching into frame. It takes a while (but not too long) to notice the *Littoraria* hiding, presumably from hot sun and predators.”

Another example of animals taking advantage of man-made habitats is illustrated in the photo by David Costantini of breeding terns making their nest on an abandoned shovel (Additional file 17). We admired the terrific composition and the matching of the colors between the shovel and the terns.

## Conservation

By exhibiting the beauty to be found in the natural world, wildlife photography can contribute to conservation efforts. ‘The roar of the last Andalusian dragon’ by Javier Ábalos Álvarez was a striking image of a charismatic lizard that is under threat from uncontrolled development (Additional file 18). Ana Carolina Lima’s image of a frog from the family Leptodactylidae certainly shows off its subject’s beauty (Additional file 19). Chris Darimont admired the “‘studio’-looking shot that works with the browns of the frog and leaf contrasted with the bright white background. I love the leaf that constructed the perch—shows how small and delicate the frog is”.

While conserving all of the earths’ biodiversity is of course crucial, charismatic flagship species will always be vital to conservation efforts. Two images showcased such iconic species in different ways. Miguel Gomez’s image of an elephant tusk reminded us of the dangers posed to their survival by the ivory trade (Additional file 20). Chris Darimont liked that the image was “framed to show enough, but not too much, of the subject, so one knows it’s an elephant. I especially appreciate the signs of wisdom, hardship and a life long-lived (furrowed skin, fractures on tusk)”. Conversely, a more cheerful portrayal of a charismatic species can be found in Arnaud Badiane’s picture of a koala peacefully relaxing between two tree branches (Additional file 21).

## The joys of the field

One reason ecology lends itself so well to this kind of competition is that many ecologists spend much of their research life working in the wild. Diogo Sayanda (who, with three images featured in our competition, clearly enjoys being out in the field) documented his rather enviable interaction with a school of dolphins (Additional file 22). Chris Darimont admired the “gorgeous colors and movement” in this photo. A slightly more startling, if no less exciting, encounter was had by Sjoerd Duijns who got surprisingly close to a polar bear during his field work in Canada’s Hudson Straight (Additional file 23).

Finally, if the idea behind our competition is to showcase the intersection between research and photography, then none of this year’s images did this more explicitly than Dewald Kleynhans’ picture ‘You better get the weight right!’ (Additional file 24). His research assesses the personalities of elephant shrews, and it’s fair to say this individual must rank towards the bold end of the spectrum. As Chris Darimont put it “he seems to be saying, ‘Is this for real?’”.

## Conclusions

We were delighted at the variety and quality of the images submitted to the 2017 Image Competition. We thank all those who took part in this year’s competition, and congratulate our winning photographers; we hope our readers enjoyed their work as much as we have. We look forward to the 2018 competition!

## Additional files

**Additional file 1.** “Sea lion resting in the coast of San Cristobal Island in Galapagos.” Attribution: Pablo Juarbe Martinez (Florida Institute of Technology, USA).

**Additional file 2.** Candle in the wind. “Epiphytic plants at flowering. An epiphyte is an organism that grows on the surface of a plant and derives its moisture and nutrients from the air, rain, water or from debris accumulating around it.” Attribution: José López Bucio (Universidad Michoacana de San Nicolás de Hidalgo, Morelia, México).

**Additional file 3.** Dicing with death. “The Malaysian orchid mantis *Hymenopus coronatus* is a predatory insect that mimics a flower blossom. They need not ambush their prey by hiding amongst flowers, instead they stand out conspicuously against vegetation and lure in their unsuspecting prey.  Bees, flies and butterflies lured in by the bright colors expect nectar rewards but instead are met by predatory strike of the waiting orchid mantis. This lucky fly lived to see another day but probably had no idea how close it came to being this mantises next meal.” Attribution: James O’Hanlon (University of New England, Australia).

**Additional file 4.** Sado River Estuary. “This is an aerial photograph of Sado River Estuary. It shows the intricate patterns shaped by the water flow and salt marshes in a constant and dynamic conflict.” Attribution: Diogo Sayanda (University of Lisbon, Portugal).

**Additional file 5.** *Atelopus hoogmoedi*. “In addition to monitoring reptile and amphibian biodiversity we also investigate homing behavior in *Atelopus hoogmoedi*. While most individuals were just sitting on their same perch site night after night, this individual took guarding his spot more seriously and looked like an actual sentinel. Either that or a lazy worker resting on its shovel.” Attribution: Michaël Nicolaï (Vrije Universiteit Brussel , Belgium).

**Additional file 6.** Mating common wall lizards. “Every year from May to July, it is the mating season for Common Wall lizards (*Podarcis muralis*). When a male, after an intense competition against rival males, can finally mate with a female, he bites her on the abdomen to prevent her escape and starts copulating. Females are often left with scars from this powerful bite.“ Attribution: Arnaud Badiane (Macquarie University, Australia).

**Additional file 7.** Sexual dimorphism. “Photograph taken in forest in French Guiana. The contrasted shape and size between a female and male spiders (*Araneidae*) on her web.” Attribution: Maïlis Huguin (Institut Pasteur de la Guyane, French Guiana).

**Additional file 8.** Multitasking dance files. “Multitasking by two dance flies (*Empididae*). The male uses only two of its six legs to hold his body and that of the female and her prey. With the other four legs he holds the female which he fertilizes. The female, while being hold, sucks blood out of a fly (probably a March fly - *Bibionidae*) which she caught just before, to supply her eggs with nutrients. The female took her time to put the prey in different positions to suck the most out of it, while the male was able to keep a stable position. What only few people know is that these predatory flies also pollinate flowers. They visit flowers regularly, and you can see pollen on the bodies of these two individuals. Will they transfer the pollen to another flower after mating?” Attribution: Jeroen Everaars (German Centre for Integrative Biodiversity Research (iDiv) Halle-Jena-Leipzig, Germany).

**Additional file 9.** “The Swallow Tailed Gull (*Creagrus furcatus*) is an endemic and common resident of the Galapagos Islands. When it is not breeding it is totally pelagic, migrating eastward to the coasts of Ecuador and Peru. The Swallow Tailed Gull is unique within the gulls for feeding exclusively at night. It is the only nocturnal gull in the world. Its night-adapted eyes allow it to feed miles from shore on fish and squid it captures from the surface of the ocean. The picture was taken in a field trip to Galapagos. A Lava Gull is about to land on the black lava rocks in the shore.” Attribution: Majoi de Novaes Nascimento (Florida Institute of Technology, USA).

**Additional file 10.** Parental care. “Several frog species carry their tadpoles to safe places. This is not easy when the wild is a huge world for you. This picture shows an *Oophaga pumilio* carrying its tadpole and looking for a safe orchid where it can put its new offspring.” Attribution: Roberto García-Roa (University of Valencia, Spain).

**Additional file 11.** Ant queen carrying larvae. “This ant queen carrying a larva is walking on colorful leaves in autumn. Ants of the species *Temnothorax crassispinus* are very small (approximately 3mm) and the whole colony lives in acorns or twigs in forests. Usually the workers care for the brood while the queen reproduces, but when the colony gets disturbed, the queen is also in action.” Attribution: Julia Giehr (University of Regensburg, Germany).

**Additional file 12.** Fish shoal. “This image shows a shoal of *Talassoma parvo* predating the eggs of *Abudefduf luridus*. Although the male desperately tries to protect its prole he can’t repel a shoal of more than 20 individuals.” Attribution: Diogo Sayanda (University of Lisbon, Portugal).

**Additional file 13.** Fungus attack. “Photograph taken in forest in French Guiana. A butterfly parasitized by a *Cordyceps* fungus in tropical primary forest.” Attribution: Maïlis Huguin (Institut Pasteur de la Guyane, French Guiana).

**Additional file 14.** *Cryptolaemus montrouzieri* larva eating mealybug runner or juvenile. “*Paracoccus marginatus* mealybug  is a serious pest of papaya. Heavy infestation in papaya may cause the fruits to become stunted. Sooty mold growing from the honeydew excreted by the mealybug can also block the sunlight and causing inefficient photosynthesis for plants. The honeydew also becomes food for ants and, in return, the ants help guard the mealybug from other predators and also parasitoids. This mealybug is hard to control with heavy infestations occurring, especially in organic farms. Surveys found that two predators and a parasite act as a biological control agent to control the mealybug. The most common predators are ladybird beetles *Chilocorus sp.* and mealybug destroyer *Cryptolaemus montrouzieri*. Parasites of the families *Braconidae* have also been recorded.” Attribution: Mohd Masri bin Saranum (Malaysian Agricultural Research and Development Institute).

**Additional file 15.** Stingless bee. “Stingless bees have become a new industry in Malaysia. Because of that, people had over collected the bees from the wild to domesticate it. This has become a major problem as stingless bees are an important pollinator, especially in the forest. To reduce the problem, conservation of the wild stingless bees is being carried out by educating people that only a few species can be domesticated. Study on how to conserve the domesticated stingless bees from pest and diseases was also carried out to sustain the industry for the long term.” Attribution: Rozilawati binti Harun (Medical Entomology Unit, Infectious Disease Research Centre, Kuala Lumpur, Malaysia).

**Additional file 16.** Periwinkles and boats in the Seychelles. “These striped periwinkles (*Littoraria coccinea*) share their habitat with people on this beach on the island of Praslin, Seychelles.  This stretch of coastline is one of the busiest on this island.” Attribution: Hannah Bose (University of Edinburgh, UK).

**Additional file 17.** Breeding terns. “Arctic terns (*Sterna paradisaea*) mate for life. They breed on the ground and both sexes share incubation duties. This photo taken in Svalbard shows that vocal communication between mates is very important to coordinate parental efforts in order to achieve a successful reproduction. But this is not all. Finding a good place where to breed may be hard in human-modified landscapes. This couple of Arctic terns found a clever solution to solve this difficult problem: they made their own house on an abandoned shovel.” Attribution: David Costantini (Museum National d’Histoire Naturelle, France).

**Additional file 18.** The roar of the last Andalusian dragon. “In southern Spain, lost in the middle of the arid interior region of Málaga, lies an area of fertile fruit plantations named the “Axarquía”. Here, the last Andalusian dragons (no other name may deserve an animal such as *Chamaeleon chamaeleo*) thrive amongst the branches of both exotic avocado and historical Mediterranean olive trees. This elusive animal may be difficult to spot throughout the year, but in summer, the striking and distinct colors signalling reproductive state in both sexes contrast with the fragility of the ecosystem it inhabits. Uncontrolled hotel building for the low-cost tourism industry in this part of the Spanish Mediterranean threatens the maintenance of healthy populations of this charismatic lizard.” Attribution: Javier Ábalos Álvarez (University of Valencia, Spain).

**Additional file 19.** Leptodactylidae. “The Leptodactylidae is just one of several families of frogs found in the Cerrado region where this picture was taken. I was in the Cantão State Park as part of a research group working in the field to collect data on the status of reptiles and amphibians’ populations. The Cerrado is the world’s most biologically rich savanna and it stretches across nearly 500 million acres of Brazil. It is one of the most unknown and unprotected savannas in the world with less than 2% of its region protected in national parks and conservation areas. Unfortunately, the expansion of large-scale agriculture across the Cerrado has endangered the biodiversity of this region and now it has become one of the most threatened and over-exploited regions in Brazil. It is of crucial importance to gather a broader knowledge and protect the species from this ecosystem.” Attribution: Ana Carolina Lima (Univeristy of Aveiro, Portugal).

**Additional file 20.** Elephant tusk. “Poaching to feed the elephant ivory trade is one of the most important threats to the conservation of this species. Elephants are one of the most charismatic animals of Africa and have been used as a flagship species for conservation efforts; conservation of elephants could mean conservation of African wild areas.” Attribution: Miguel Gomez (University of Manchester, UK).

**Additional file 21.** Peaceful koala. “This picture of a Koala sitting on a branch was taken around Canberra in Australia. Koalas are considered as vulnerable by the IUCN Red List of species and are subject to conservation programs in Australia.” Attribution: Arnaud Badiane (Macquarie University, Sydney, Australia).

**Additional file 22.** Dolphin school. “This photograph represents a part of a >100 individuals group of common dolphins (*Delphinus delphis*) in the oceanic waters of Madeira. It is difficult to describe the experience of being among these animals in their natural environment, everything is chaotic they swim around you, jump, the females approach you with their calves, it’s an overwhelming amazing experience.” Attribution: Diogo Sayanda (University of Lisbon, Portugal).

**Additional file 23.** Polar bear. “Polar bear predation of common eider nests seems to be increasing because of sea ice loss. We are working to understand how sea ice loss is influencing polar bear foraging behaviour, and what effects this might have on common eider duck populations. This research is being done by boat-based surveys in the Hudson Strait, east of Cape Dorset in northern Canada, in close collaboration with local Inuit guides and assistants. On pre-surveyed islands, we counted the number of active and destroyed common eider nests, to quantify numbers of nest and their breeding success. We also recorded any polar bear signs, such as scat, footprints, and destroyed nests to quantify polar bear abundance. This image highlights the unpredictable nature of our work and sometimes you encounter a face to face with a giant male polar bear scavenging for eider eggs.” Attribution: Sjoerd Duijns (Carleton University, Canada).

**Additional file 24.** You better get the weight right! “I assisted with research on the behaviour of elephant shrews (*Elephantulus myurus*) trying to determine how personalities might affect their ecology. These animals have vastly different personalities, ranging from extremely aggressive to timid and some individuals are quite bold, as is the case for this individual, who is curiously exploring the research station, making sure its body measurements have been correctly recorded.” Attribution: Dewald Kleynhans (University of Pretoria, South Africa).
