# Supplementary figures and images for: BMC ecology image competition 2017: the winning images
Source: BMC Ecol. 2017 Aug 18;17:28. doi: 10.1186/s12898-017-0138-8 (PMC5561582; doi:10.1186/s12898-017-0138-8)

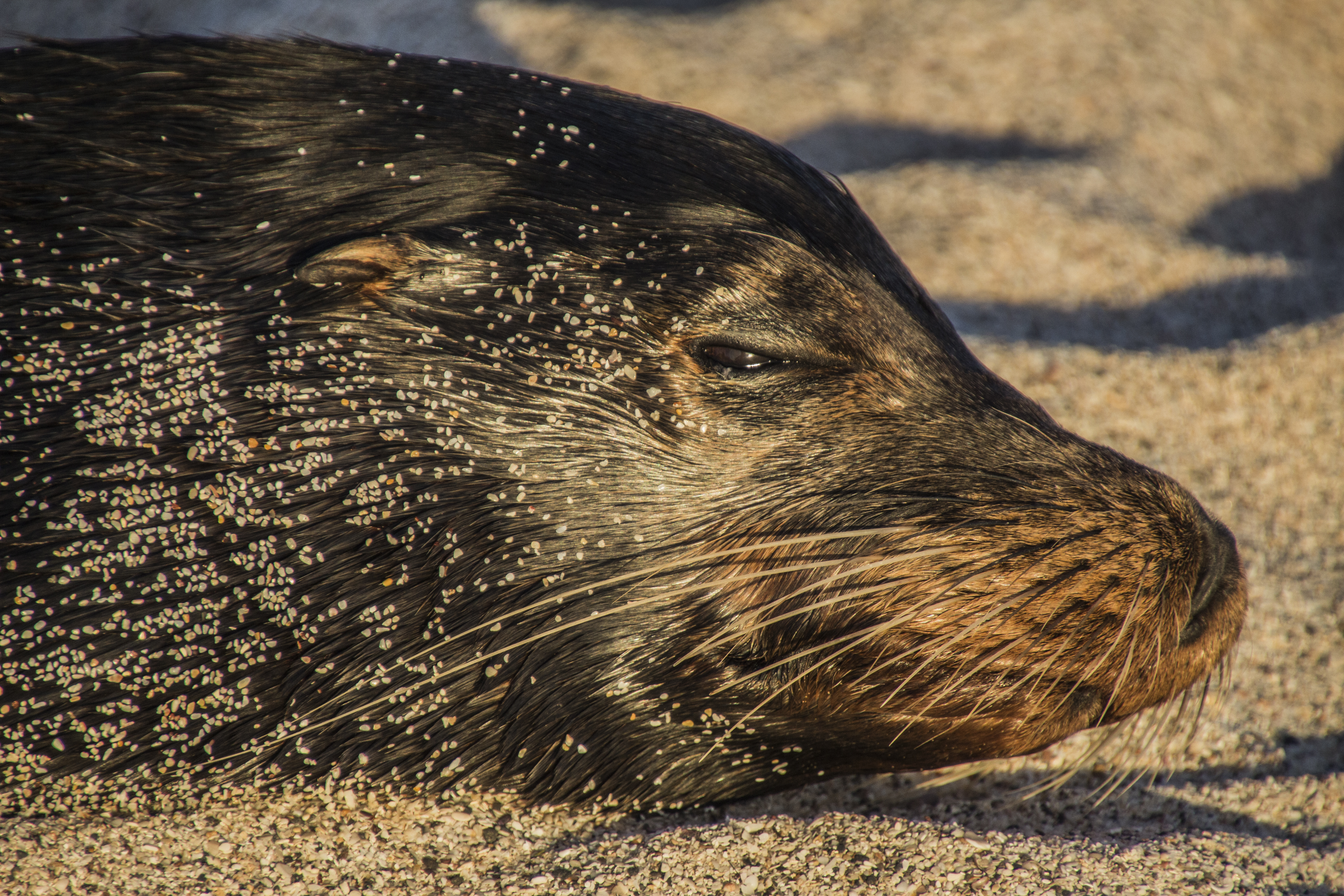

Supplement: Supplementary file 1 — Additional file 1. “Sea lion resting in the coast of San Cristobal Island in Galapagos.” Attribution: Pablo Juarbe Martinez (Florida Institute of Technology, USA). [file 12898_2017_138_MOESM1_ESM.jpg]

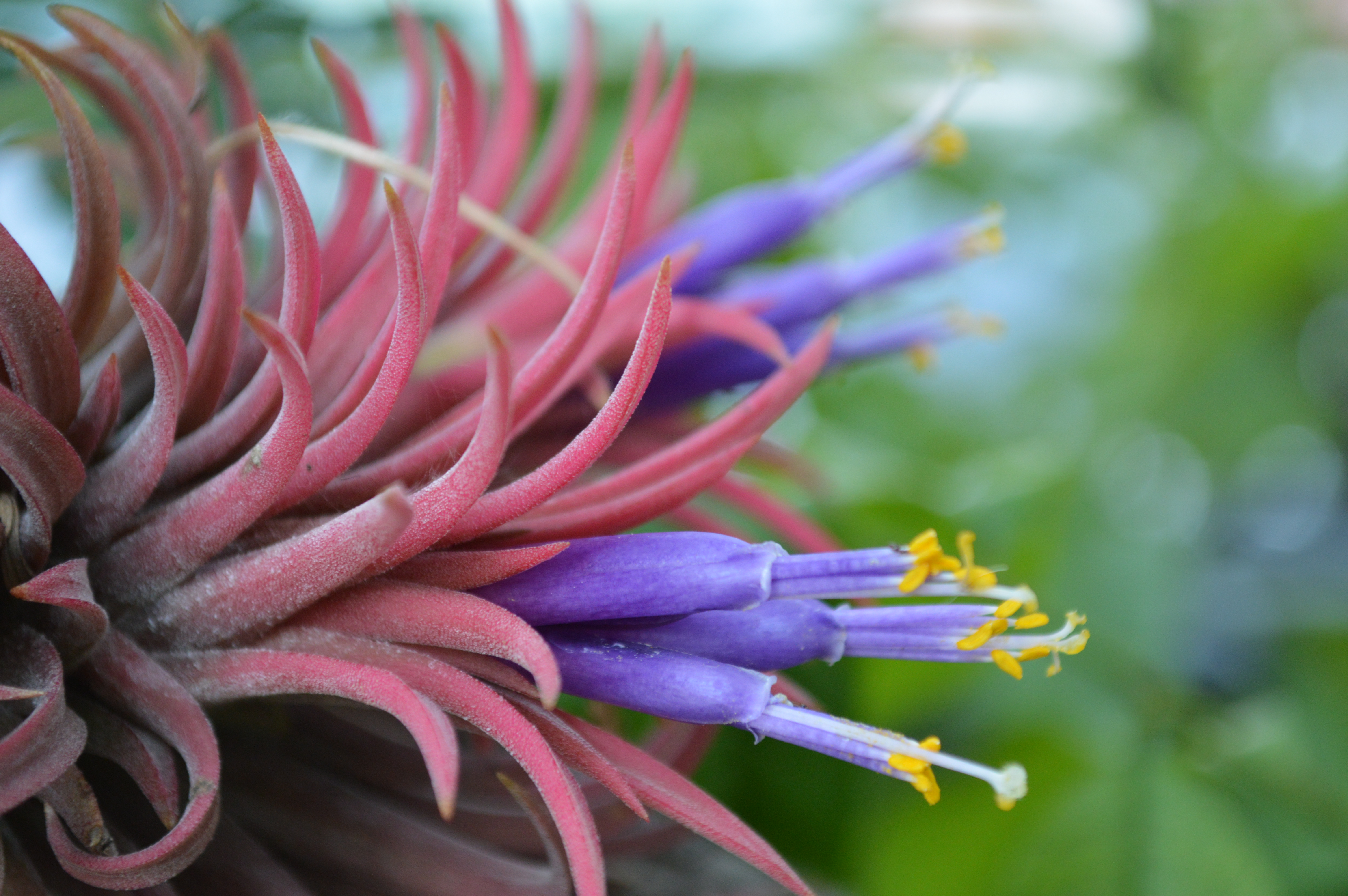

Supplement: Supplementary file 2 — Additional file 2. Candle in the wind. “Epiphytic plants at flowering. An epiphyte is an organism that grows on the surface of a plant and derives its moisture and nutrients from the air, rain, water or from debris accumulating around it.” Attribution: José López Bucio (Universidad Michoacana de San Nicolás de Hidalgo, Morelia, México). [file 12898_2017_138_MOESM2_ESM.jpg]

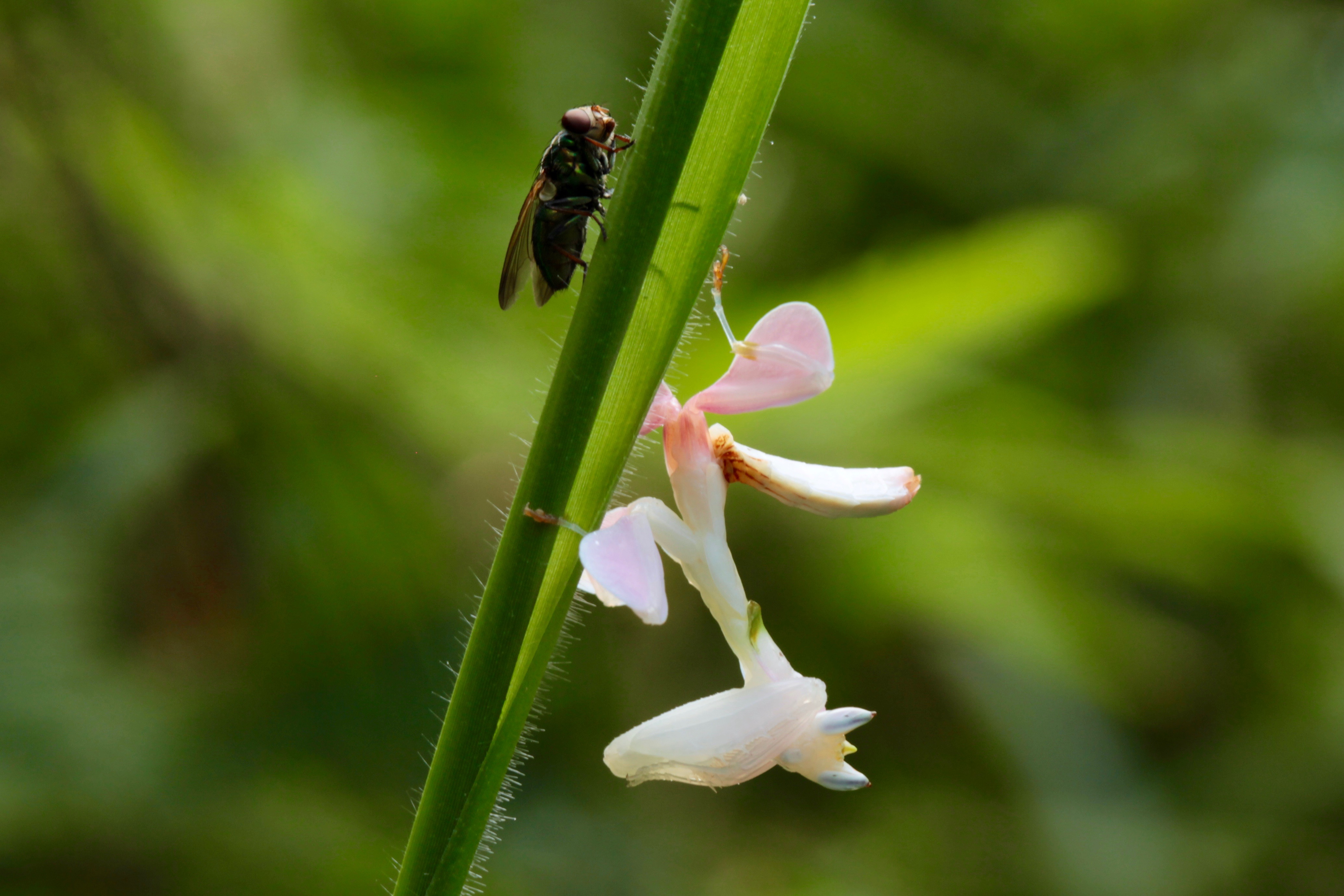

Supplement: Supplementary file 3 — Additional file 3. Dicing with death. “The Malaysian orchid mantis Hymenopus coronatus is a predatory insect that mimics a flower blossom. They need not ambush their prey by hiding amongst flowers, instead they stand out conspicuously against vegetation and lure in their unsuspecting prey. Bees, flies and butterflies lured in by the bright colors expect nectar rewards but instead are met by predatory strike of the waiting orchid mantis. This lucky fly lived to see another day but probably had no idea how close it came to being this mantises next meal.” Attribution: James O’Hanlon (University of New England, Australia). [file 12898_2017_138_MOESM3_ESM.jpg]

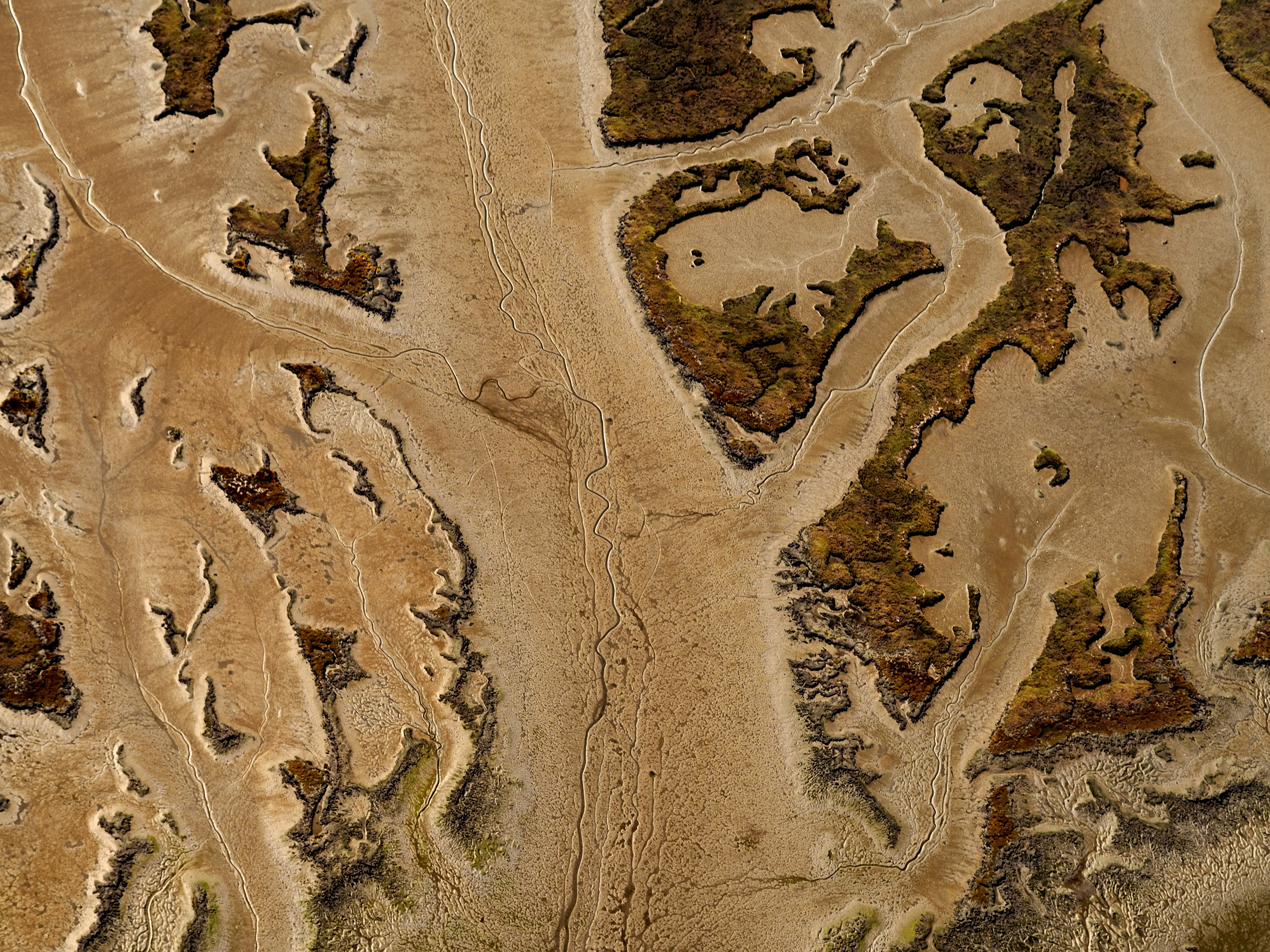

Supplement: Supplementary file 4 — Additional file 4. Sado River Estuary. “This is an aerial photograph of Sado River Estuary. It shows the intricate patterns shaped by the water flow and salt marshes in a constant and dynamic conflict.” Attribution: Diogo Sayanda (University of Lisbon, Portugal). [file 12898_2017_138_MOESM4_ESM.jpg]

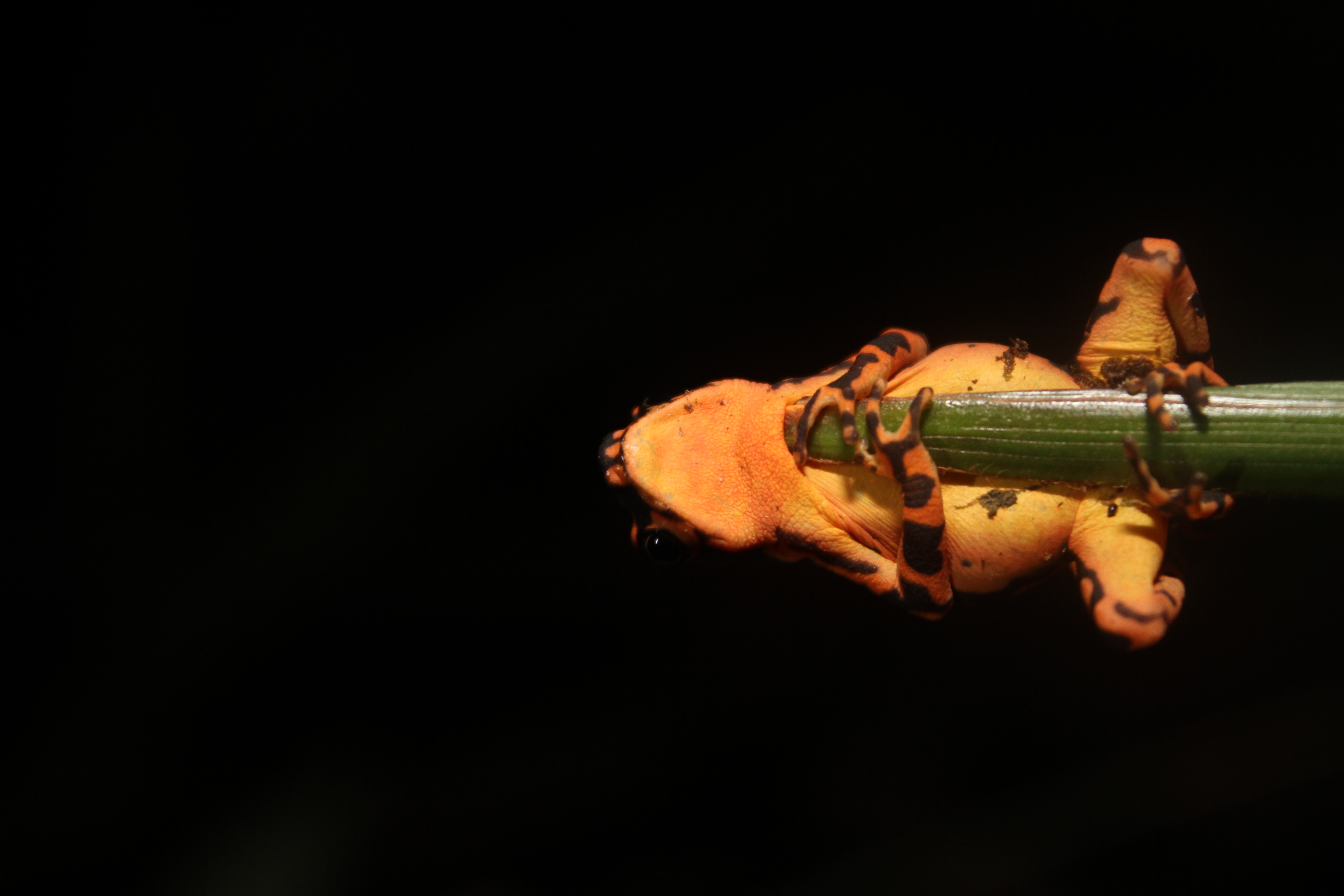

Supplement: Supplementary file 5 — Additional file 5. Atelopus hoogmoedi. “In addition to monitoring reptile and amphibian biodiversity we also investigate homing behavior in Atelopus hoogmoedi. While most individuals were just sitting on their same perch site night after night, this individual took guarding his spot more seriously and looked like an actual sentinel. Either that or a lazy worker resting on its shovel.” Attribution: Michaël Nicolaï (Vrije Universiteit Brussel , Belgium). [file 12898_2017_138_MOESM5_ESM.jpg]

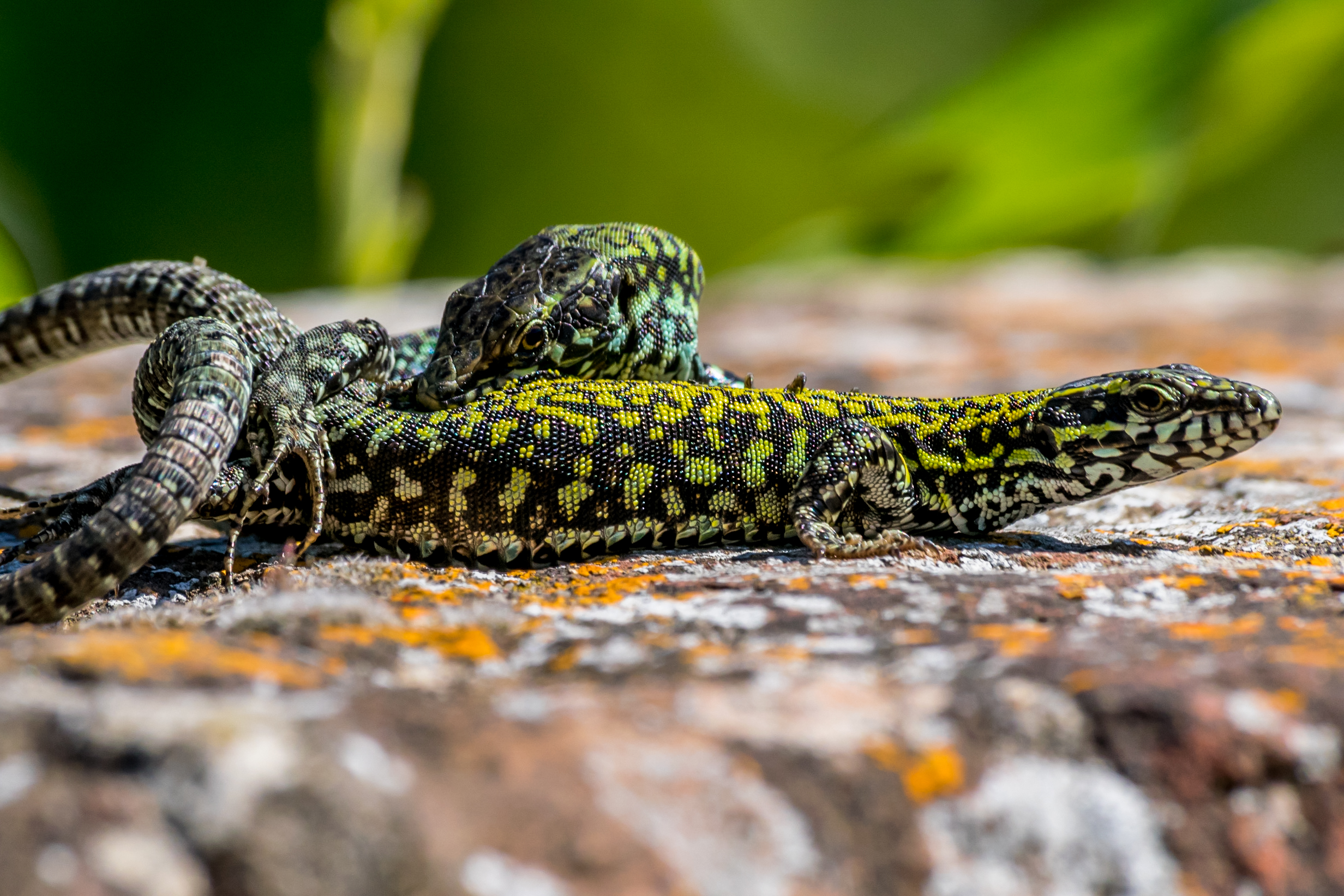

Supplement: Supplementary file 6 — Additional file 6. Mating common wall lizards. “Every year from May to July, it is the mating season for Common Wall lizards (Podarcis muralis). When a male, after an intense competition against rival males, can finally mate with a female, he bites her on the abdomen to prevent her escape and starts copulating. Females are often left with scars from this powerful bite.“ Attribution: Arnaud Badiane (Macquarie University, Australia). [file 12898_2017_138_MOESM6_ESM.jpg]

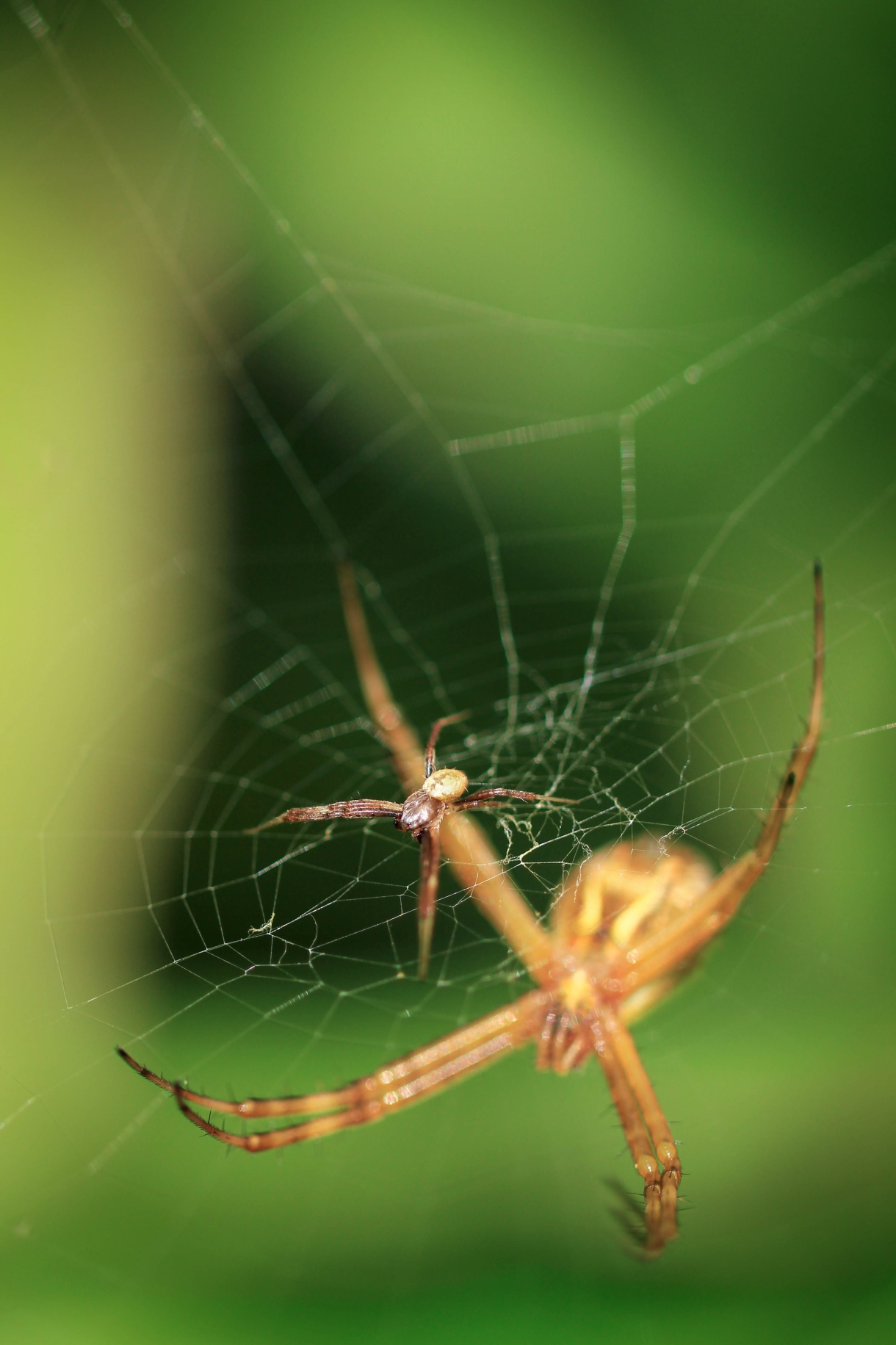

Supplement: Supplementary file 7 — Additional file 7. Sexual dimorphism. “Photograph taken in forest in French Guiana. The contrasted shape and size between a female and male spiders (Araneidae) on her web.” Attribution: Maïlis Huguin (Institut Pasteur de la Guyane, French Guiana). [file 12898_2017_138_MOESM7_ESM.jpg]

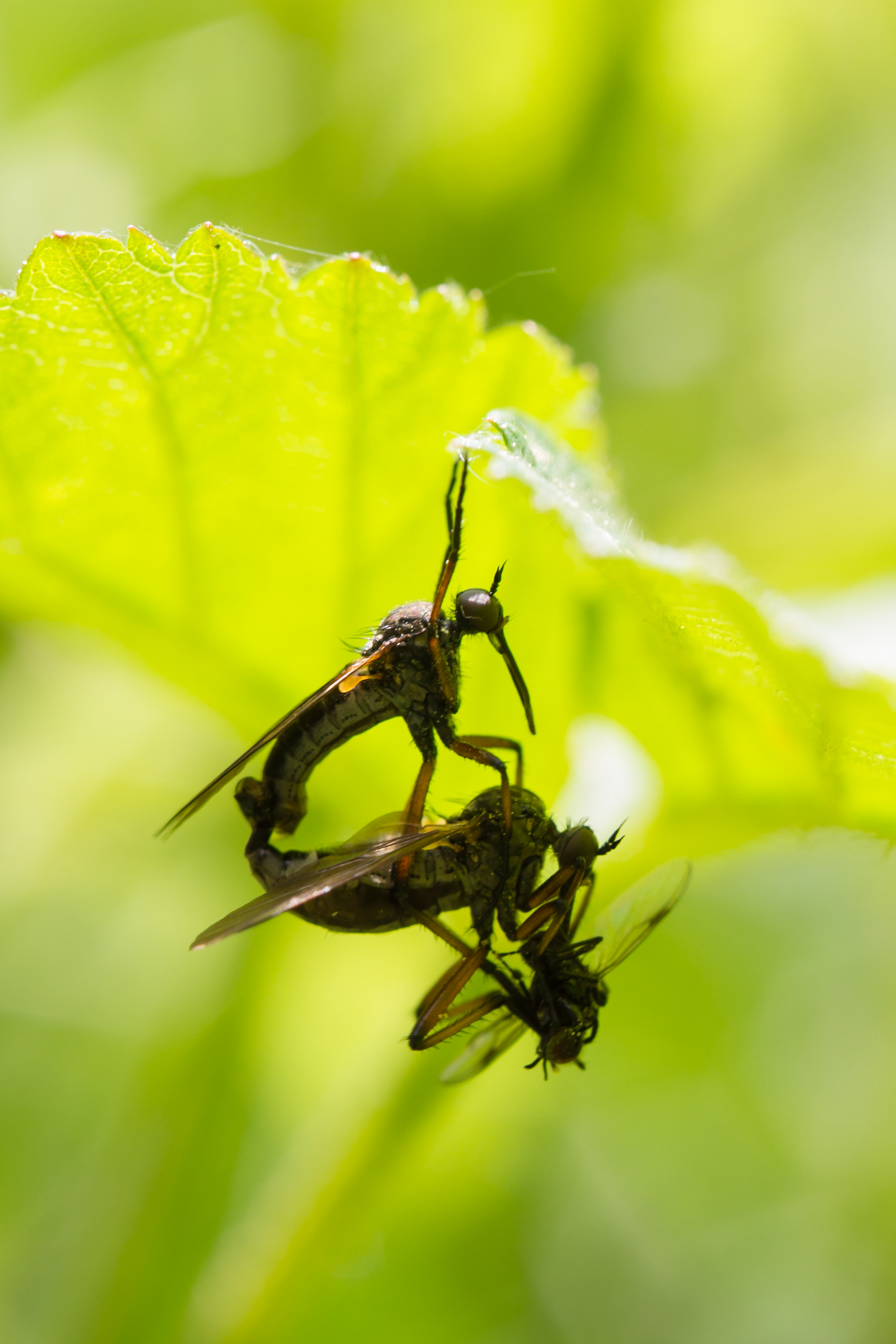

Supplement: Supplementary file 8 — Additional file 8. Multitasking dance files. “Multitasking by two dance flies (Empididae). The male uses only two of its six legs to hold his body and that of the female and her prey. With the other four legs he holds the female which he fertilizes. The female, while being hold, sucks blood out of a fly (probably a March fly - Bibionidae) which she caught just before, to supply her eggs with nutrients. The female took her time to put the prey in different positions to suck the most out of it, while the male was able to keep a stable position. What only few people know is that these predatory flies also pollinate flowers. They visit flowers regularly, and you can see pollen on the bodies of these two individuals. Will they transfer the pollen to another flower after mating?” Attribution: Jeroen Everaars (German Centre for Integrative Biodiversity Research (iDiv) Halle-Jena-Leipzig, Germany). [file 12898_2017_138_MOESM8_ESM.jpg]

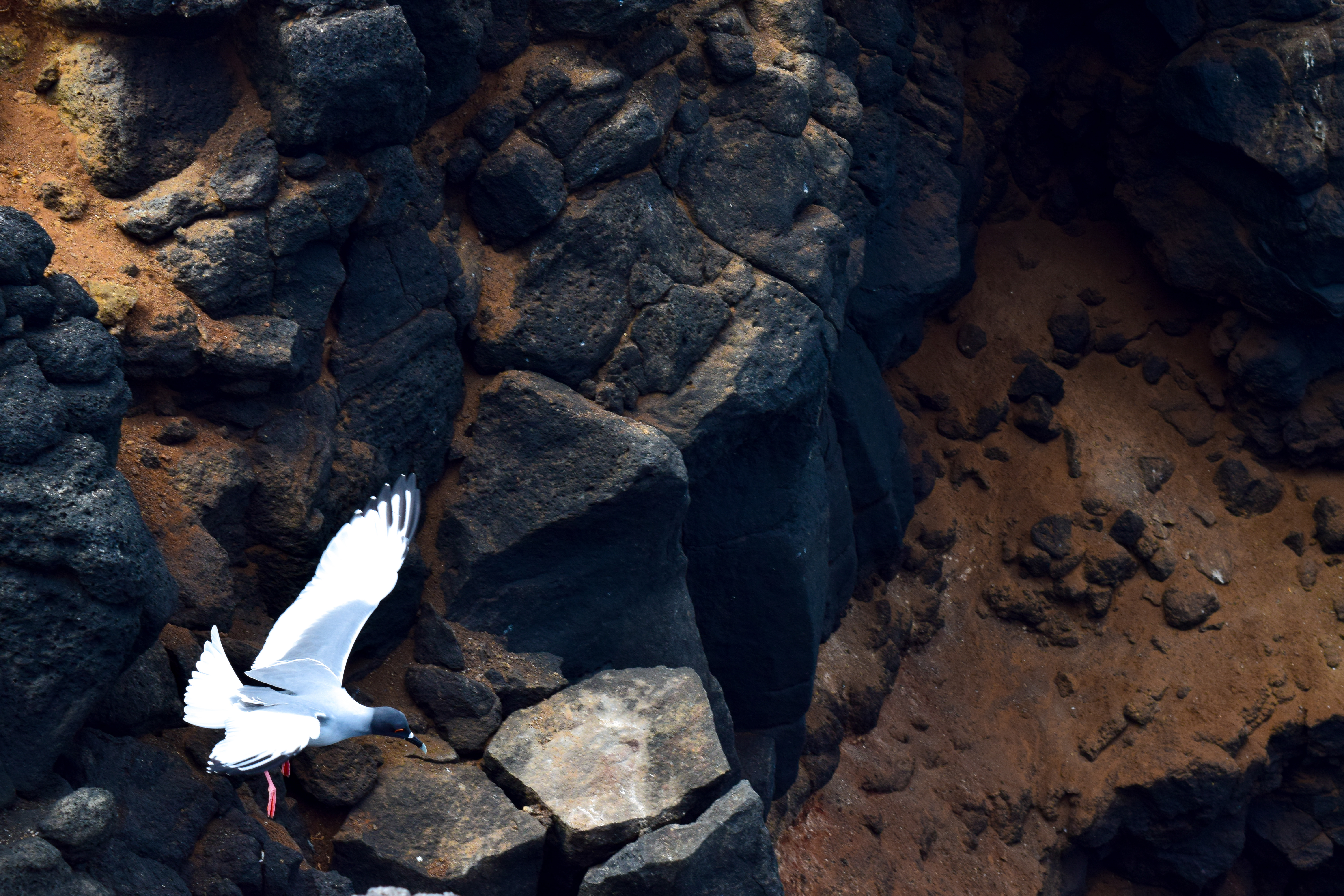

Supplement: Supplementary file 9 — Additional file 9. “The Swallow Tailed Gull (Creagrus furcatus) is an endemic and common resident of the Galapagos Islands. When it is not breeding it is totally pelagic, migrating eastward to the coasts of Ecuador and Peru. The Swallow Tailed Gull is unique within the gulls for feeding exclusively at night. It is the only nocturnal gull in the world. Its night-adapted eyes allow it to feed miles from shore on fish and squid it captures from the surface of the ocean. The picture was taken in a field trip to Galapagos. A Lava Gull is about to land on the black lava rocks in the shore.” Attribution: Majoi de Novaes Nascimento (Florida Institute of Technology, USA). [file 12898_2017_138_MOESM9_ESM.jpg]

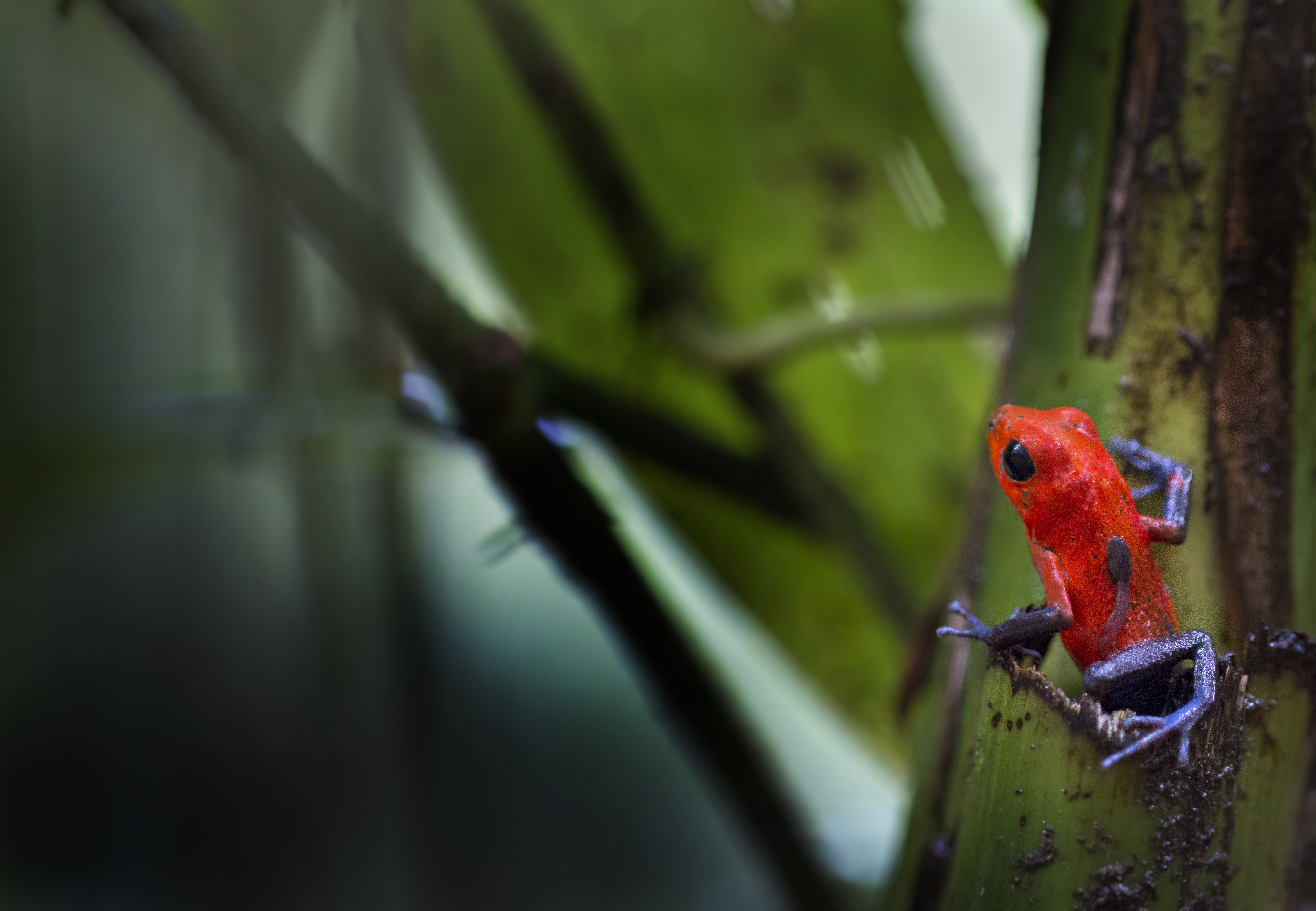

Supplement: Supplementary file 10 — Additional file 10. Parental care. “Several frog species carry their tadpoles to safe places. This is not easy when the wild is a huge world for you. This picture shows an Oophaga pumilio carrying its tadpole and looking for a safe orchid where it can put its new offspring.” Attribution: Roberto García-Roa (University of Valencia, Spain). [file 12898_2017_138_MOESM10_ESM.tif]

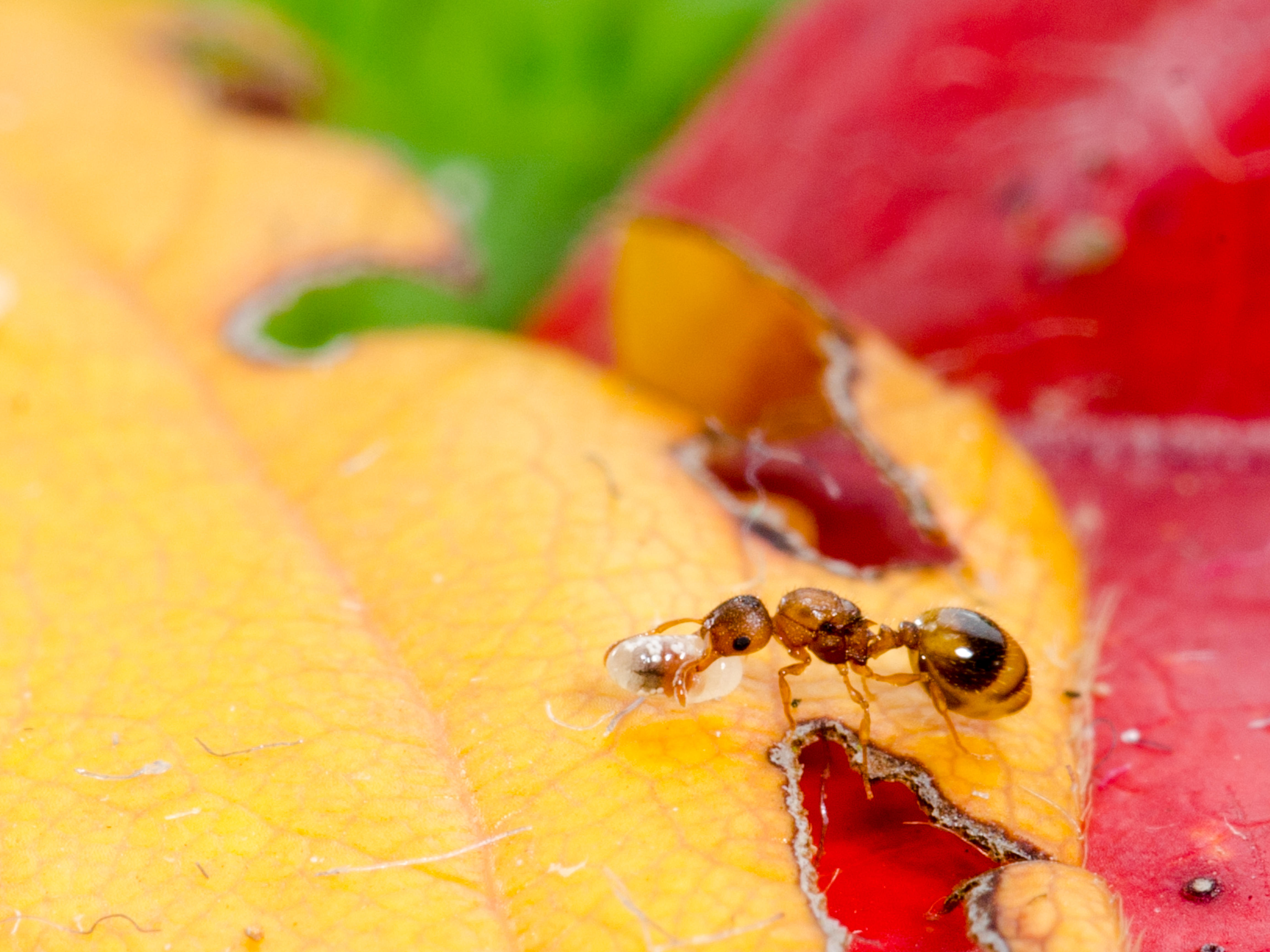

Supplement: Supplementary file 11 — Additional file 11. Ant queen carrying larvae. “This ant queen carrying a larva is walking on colorful leaves in autumn. Ants of the species Temnothorax crassispinus are very small (approximately 3mm) and the whole colony lives in acorns or twigs in forests. Usually the workers care for the brood while the queen reproduces, but when the colony gets disturbed, the queen is also in action.” Attribution: Julia Giehr (University of Regensburg, Germany). [file 12898_2017_138_MOESM11_ESM.jpg]

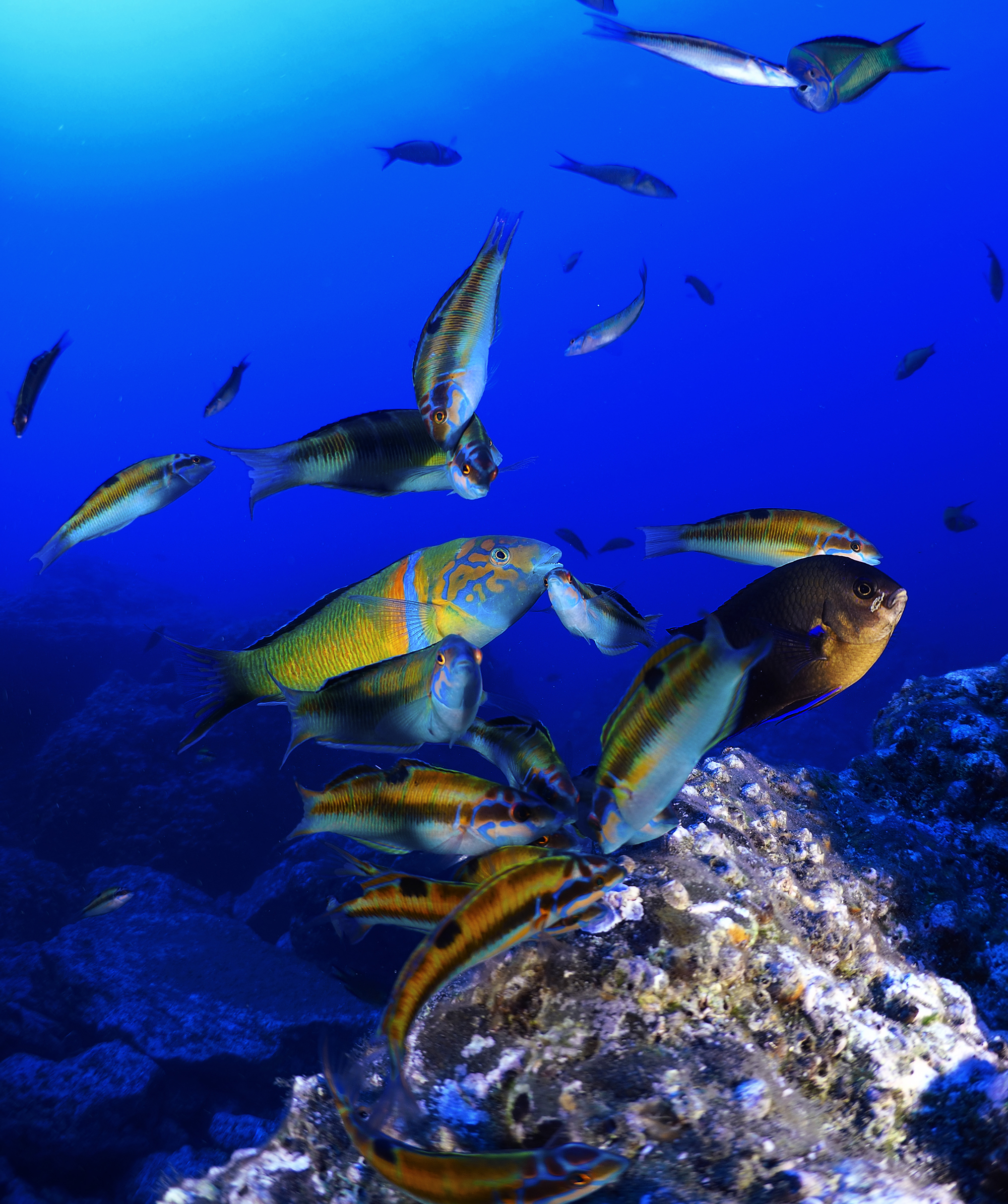

Supplement: Supplementary file 12 — Additional file 12. Fish shoal. “This image shows a shoal of Talassoma parvo predating the eggs of Abudefduf luridus. Although the male desperately tries to protect its prole he can’t repel a shoal of more than 20 individuals.” Attribution: Diogo Sayanda (University of Lisbon, Portugal). [file 12898_2017_138_MOESM12_ESM.jpg]

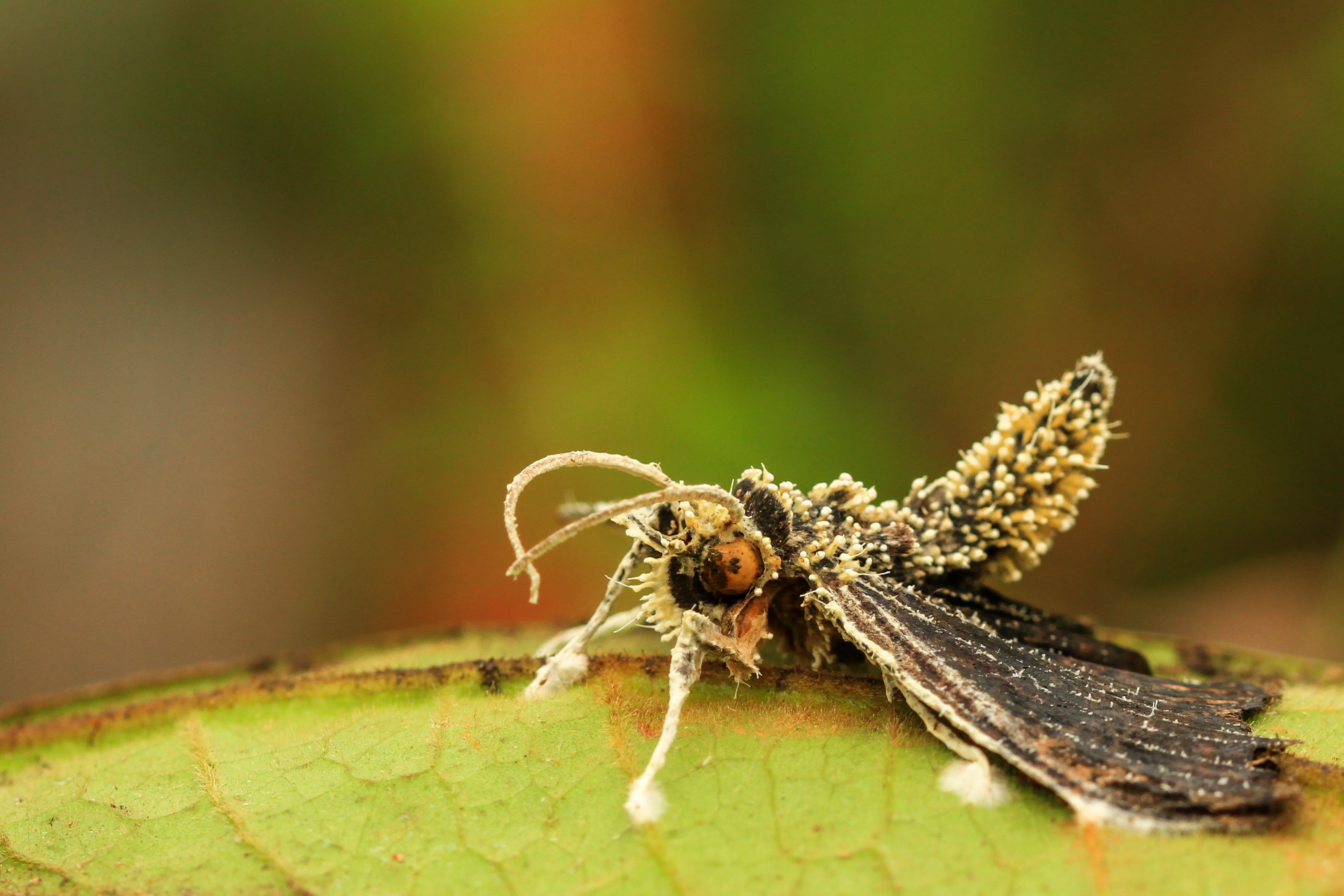

Supplement: Supplementary file 13 — Additional file 13. Fungus attack. “Photograph taken in forest in French Guiana. A butterfly parasitized by a Cordyceps fungus in tropical primary forest.” Attribution: Maïlis Huguin (Institut Pasteur de la Guyane, French Guiana). [file 12898_2017_138_MOESM13_ESM.jpg]

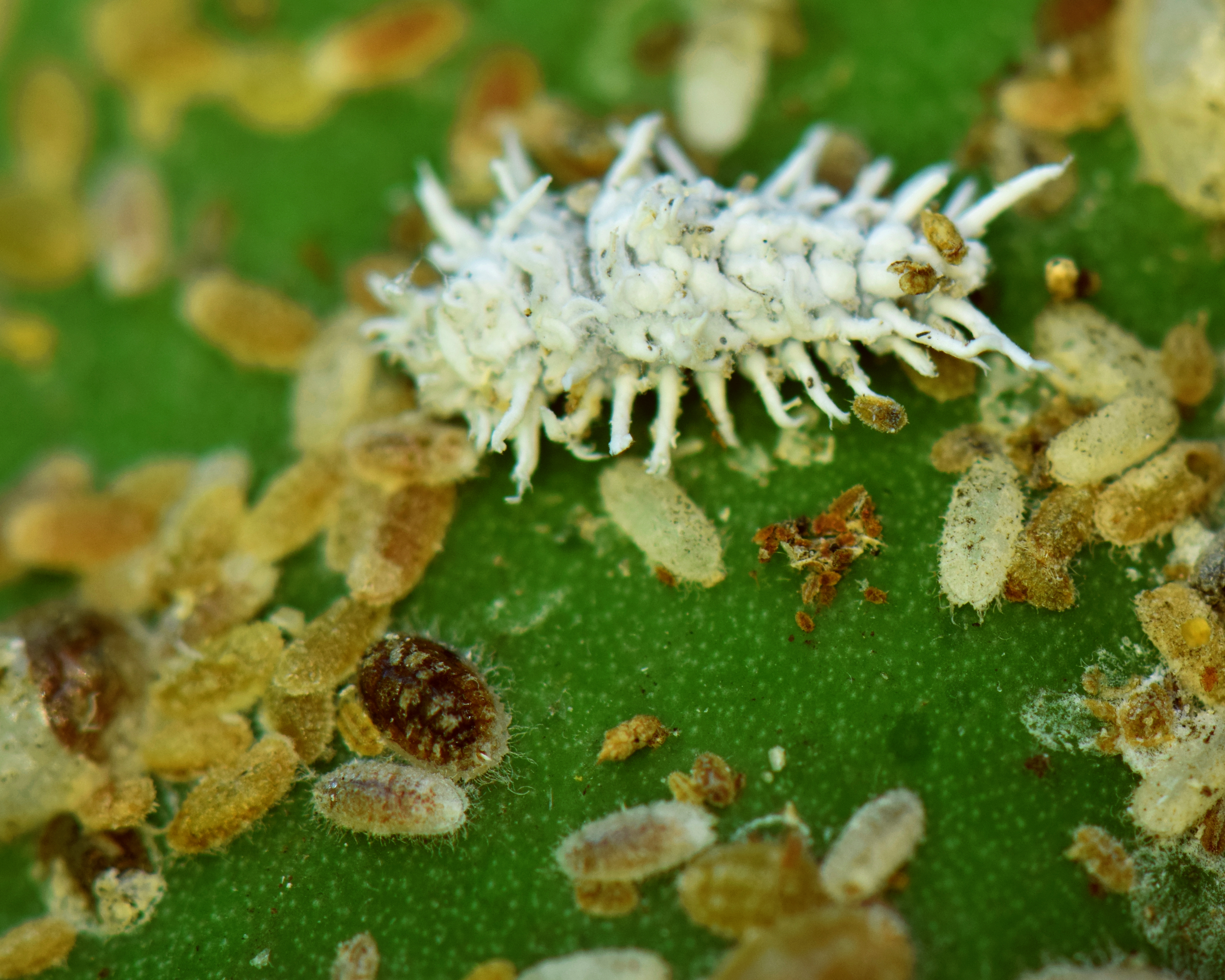

Supplement: Supplementary file 14 — Additional file 14. Cryptolaemus montrouzieri larva eating mealybug runner or juvenile. “Paracoccus marginatus mealybug is a serious pest of papaya. Heavy infestation in papaya may cause the fruits to become stunted. Sooty mold growing from the honeydew excreted by the mealybug can also block the sunlight and causing inefficient photosynthesis for plants. The honeydew also becomes food for ants and, in return, the ants help guard the mealybug from other predators and also parasitoids. This mealybug is hard to control with heavy infestations occurring, especially in organic farms. Surveys found that two predators and a parasite act as a biological control agent to control the mealybug. The most common predators are ladybird beetles Chilocorus sp. and mealybug destroyer Cryptolaemus montrouzieri. Parasites of the families Braconidae have also been recorded.” Attribution: Mohd Masri bin Saranum (Malaysian Agricultural Research and Development Institute). [file 12898_2017_138_MOESM14_ESM.jpg]

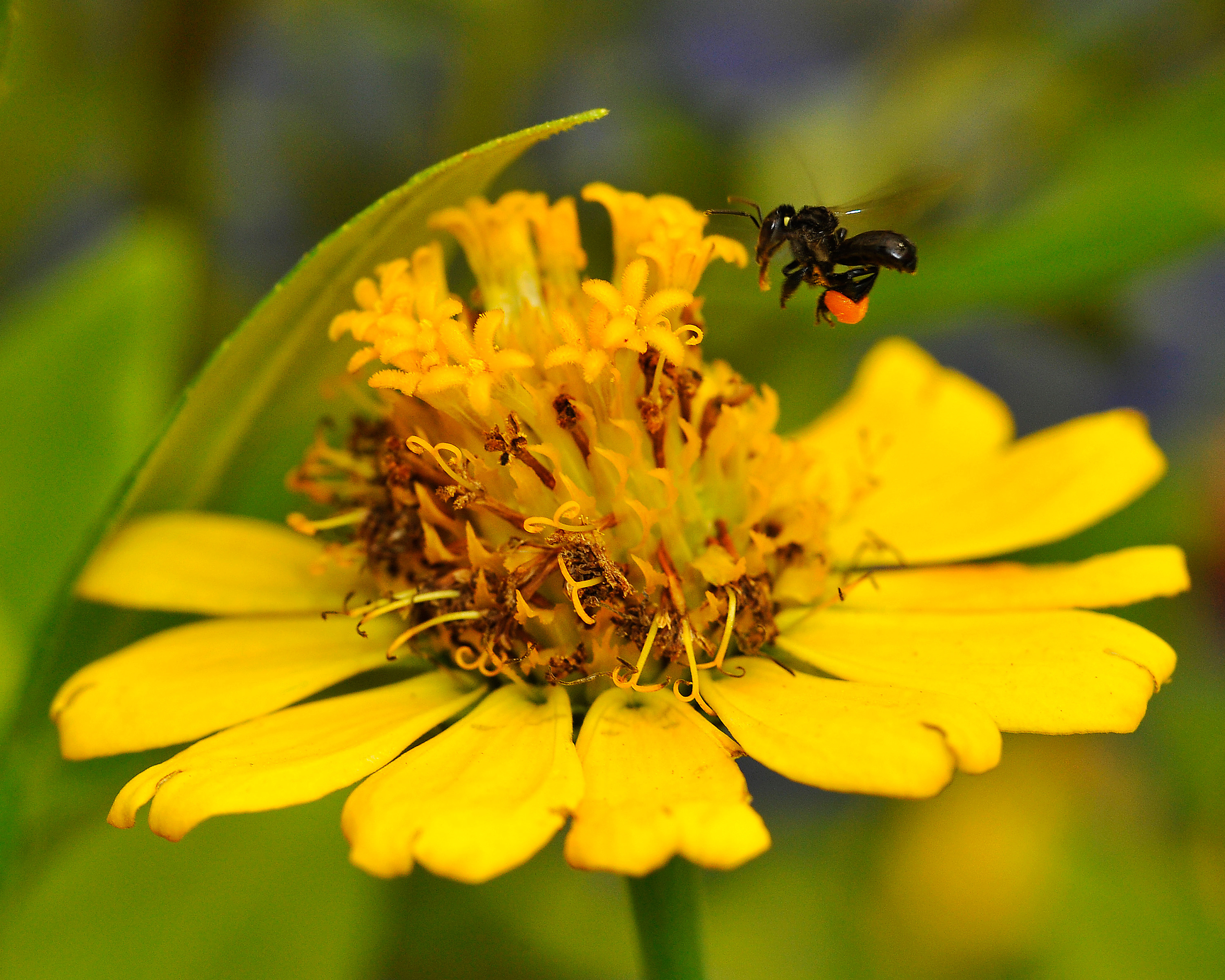

Supplement: Supplementary file 15 — Additional file 15. Stingless bee. “Stingless bees have become a new industry in Malaysia. Because of that, people had over collected the bees from the wild to domesticate it. This has become a major problem as stingless bees are an important pollinator, especially in the forest. To reduce the problem, conservation of the wild stingless bees is being carried out by educating people that only a few species can be domesticated. Study on how to conserve the domesticated stingless bees from pest and diseases was also carried out to sustain the industry for the long term.” Attribution: Rozilawati binti Harun (Medical Entomology Unit, Infectious Disease Research Centre, Kuala Lumpur, Malaysia). [file 12898_2017_138_MOESM15_ESM.jpg]

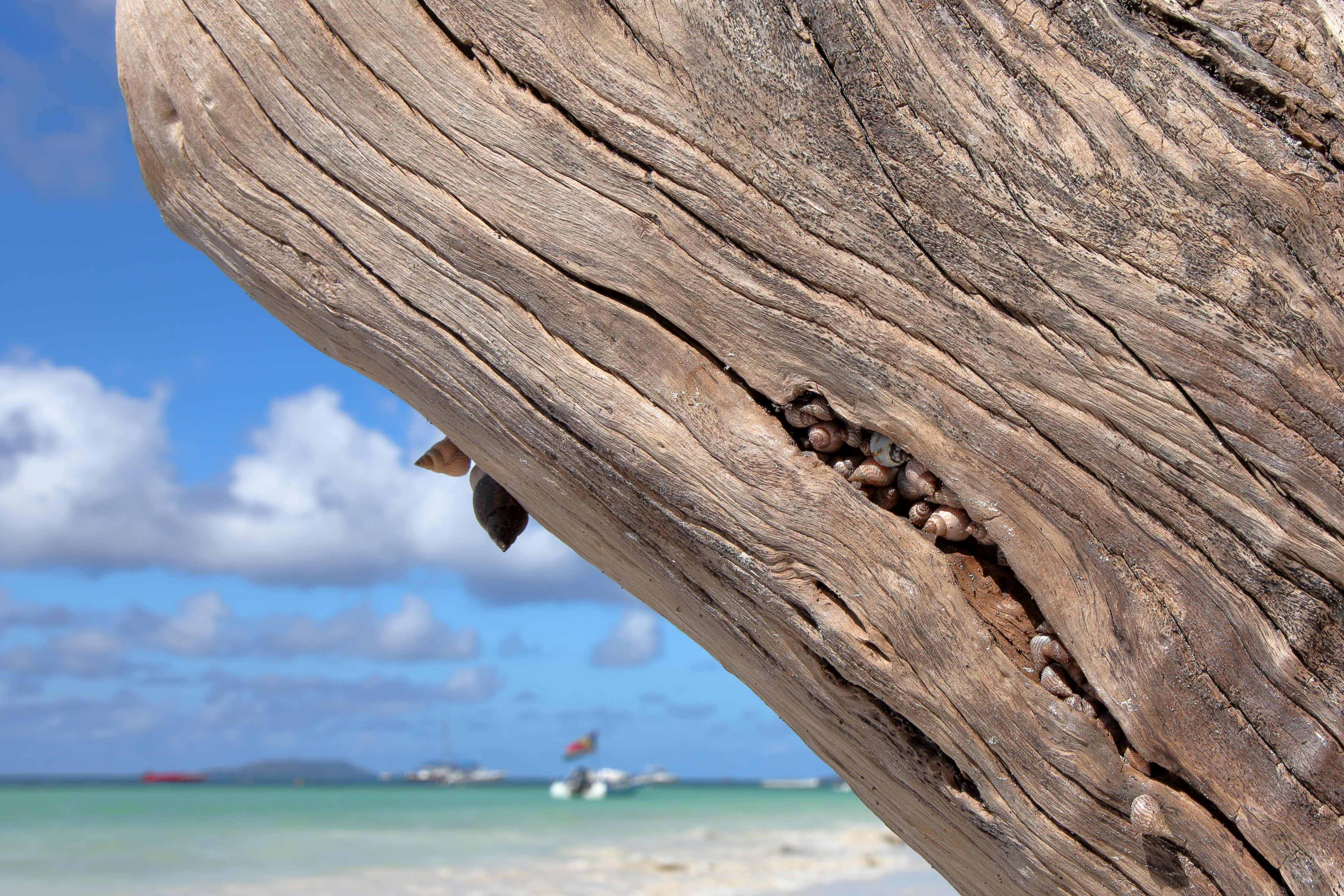

Supplement: Supplementary file 16 — Additional file 16. Periwinkles and boats in the Seychelles. “These striped periwinkles (Littoraria coccinea) share their habitat with people on this beach on the island of Praslin, Seychelles. This stretch of coastline is one of the busiest on this island.” Attribution: Hannah Bose (University of Edinburgh, UK). [file 12898_2017_138_MOESM16_ESM.jpg]

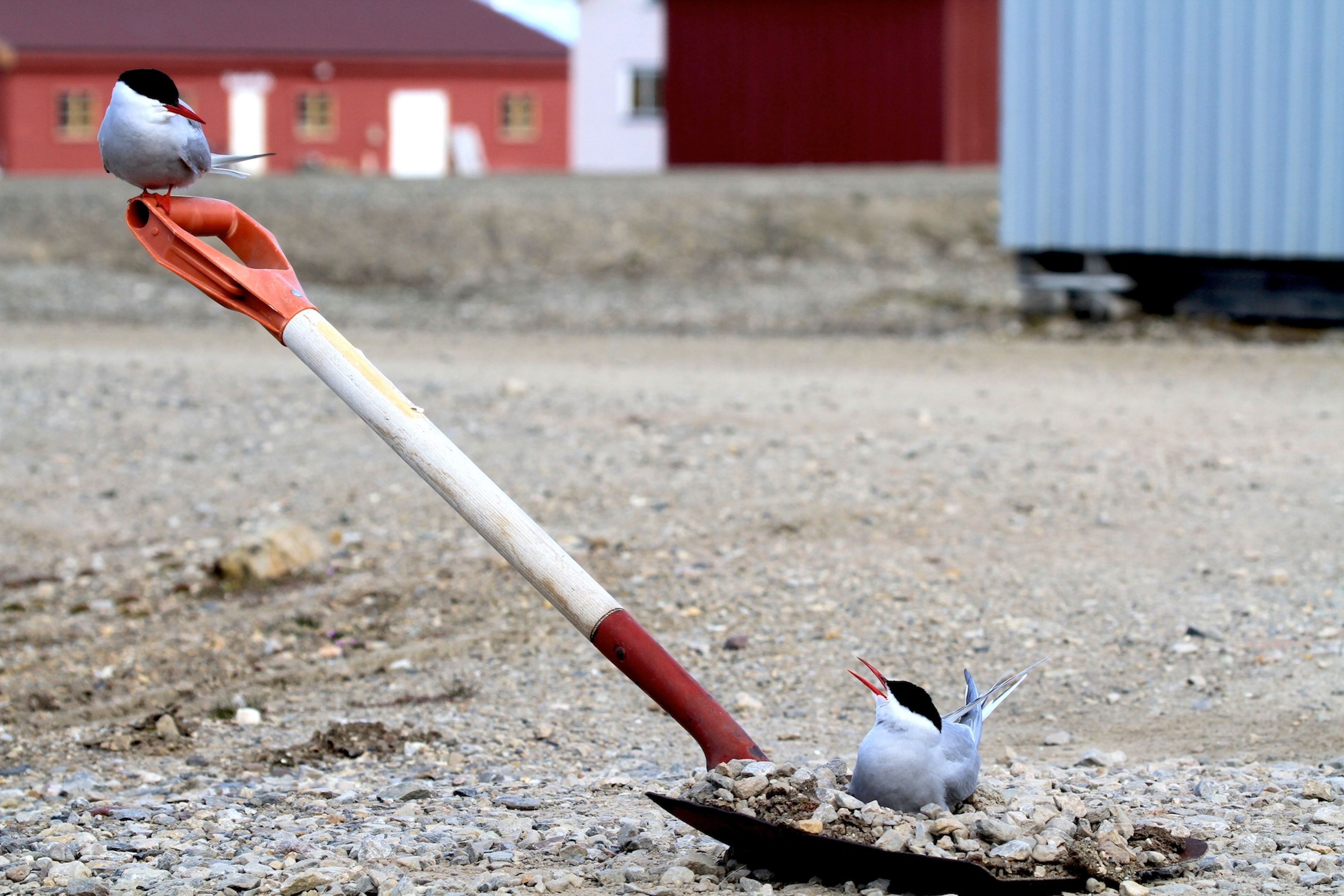

Supplement: Supplementary file 17 — Additional file 17. Breeding terns. “Arctic terns (Sterna paradisaea) mate for life. They breed on the ground and both sexes share incubation duties. This photo taken in Svalbard shows that vocal communication between mates is very important to coordinate parental efforts in order to achieve a successful reproduction. But this is not all. Finding a good place where to breed may be hard in human-modified landscapes. This couple of Arctic terns found a clever solution to solve this difficult problem: they made their own house on an abandoned shovel.” Attribution: David Costantini (Museum National d’Histoire Naturelle, France). [file 12898_2017_138_MOESM17_ESM.jpg]

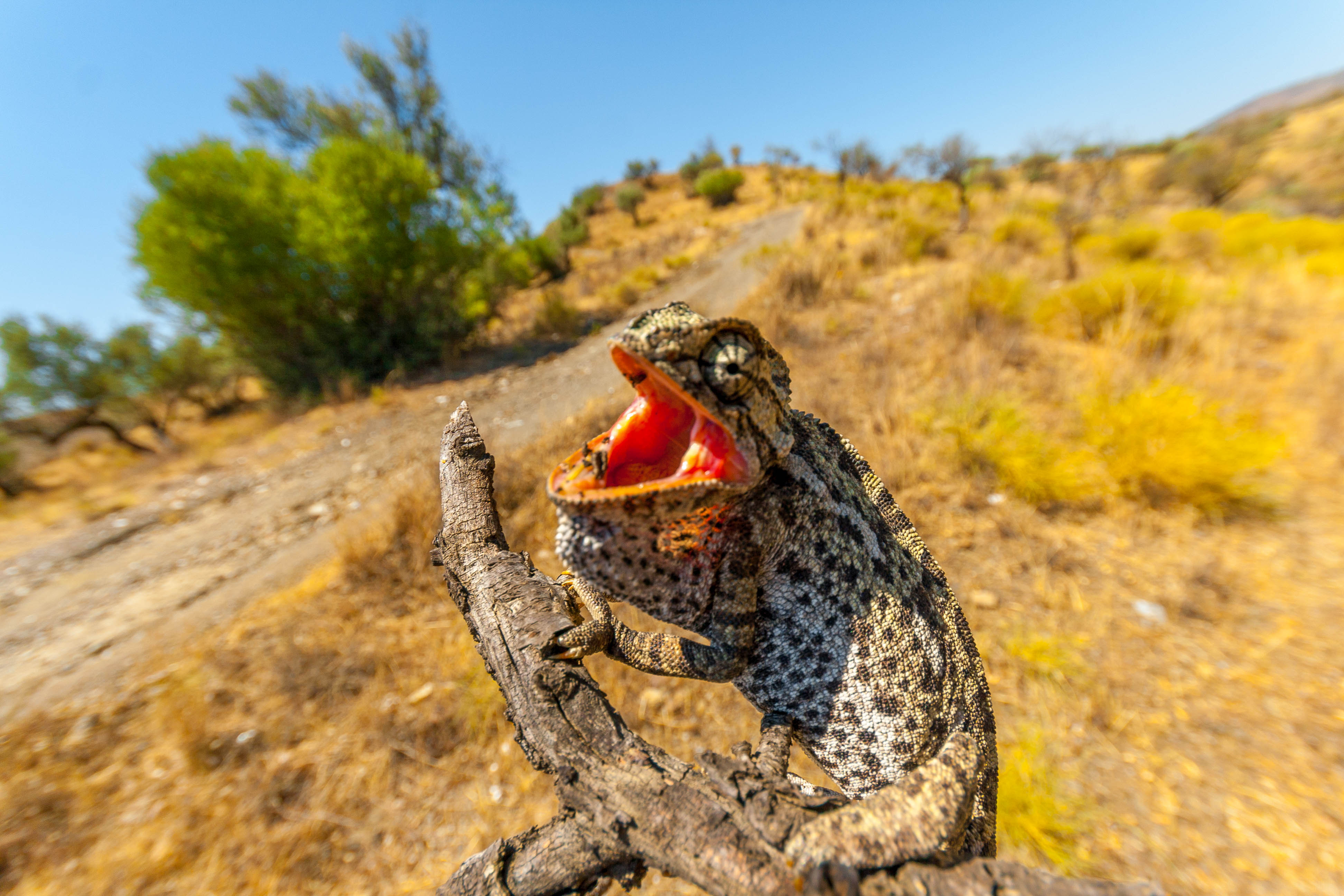

Supplement: Supplementary file 18 — Additional file 18. The roar of the last Andalusian dragon. “In southern Spain, lost in the middle of the arid interior region of Málaga, lies an area of fertile fruit plantations named the “Axarquía”. Here, the last Andalusian dragons (no other name may deserve an animal such as Chamaeleon chamaeleo) thrive amongst the branches of both exotic avocado and historical Mediterranean olive trees. This elusive animal may be difficult to spot throughout the year, but in summer, the striking and distinct colors signalling reproductive state in both sexes contrast with the fragility of the ecosystem it inhabits. Uncontrolled hotel building for the low-cost tourism industry in this part of the Spanish Mediterranean threatens the maintenance of healthy populations of this charismatic lizard.” Attribution: Javier Ábalos Álvarez (University of Valencia, Spain). [file 12898_2017_138_MOESM18_ESM.jpg]

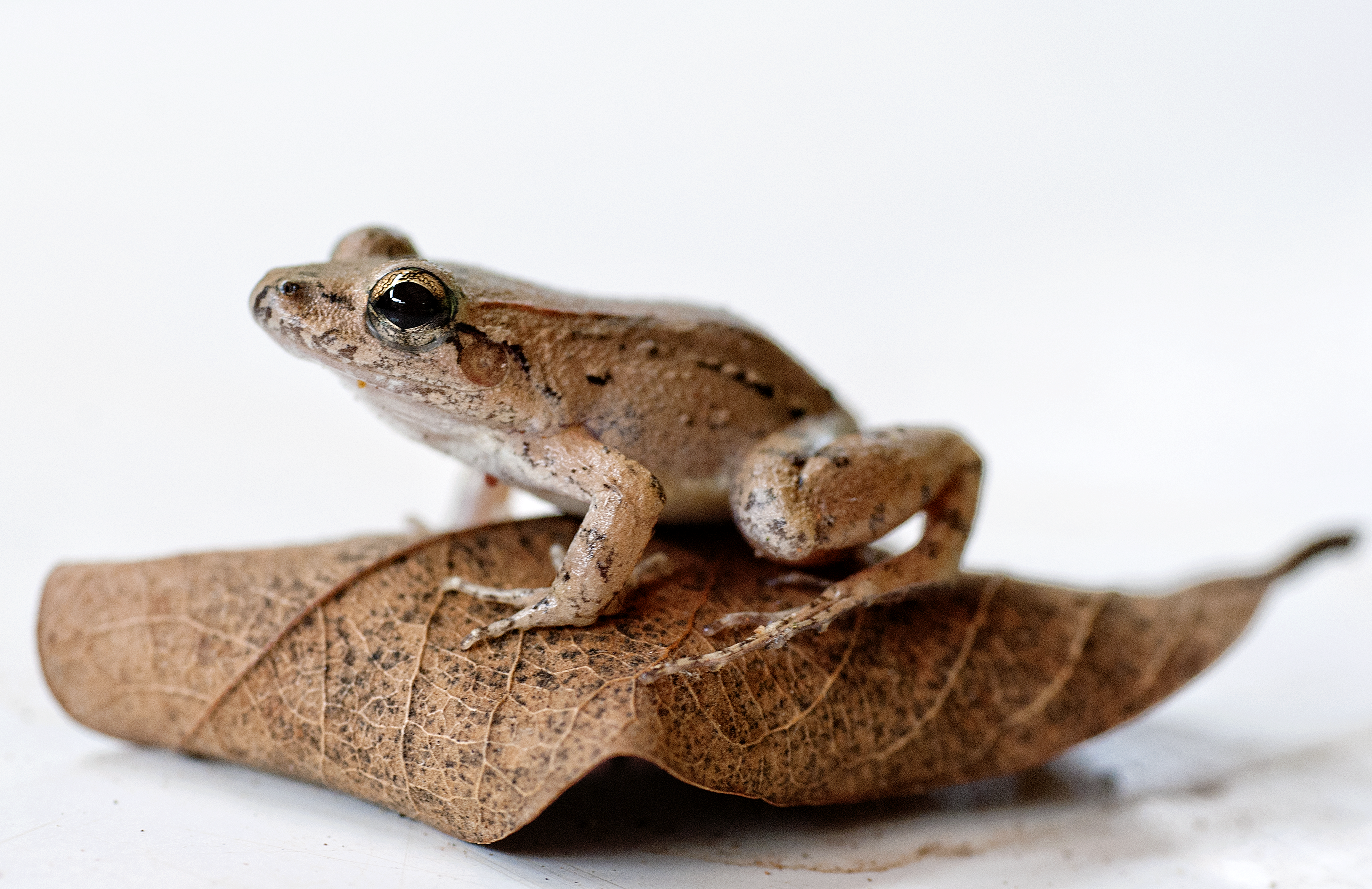

Supplement: Supplementary file 19 — Additional file 19. Leptodactylidae. “The Leptodactylidae is just one of several families of frogs found in the Cerrado region where this picture was taken. I was in the Cantão State Park as part of a research group working in the field to collect data on the status of reptiles and amphibians’ populations. The Cerrado is the world’s most biologically rich savanna and it stretches across nearly 500 million acres of Brazil. It is one of the most unknown and unprotected savannas in the world with less than 2% of its region protected in national parks and conservation areas. Unfortunately, the expansion of large-scale agriculture across the Cerrado has endangered the biodiversity of this region and now it has become one of the most threatened and over-exploited regions in Brazil. It is of crucial importance to gather a broader knowledge and protect the species from this ecosystem.” Attribution: Ana Carolina Lima (Univeristy of Aveiro, Portugal). [file 12898_2017_138_MOESM19_ESM.tif]

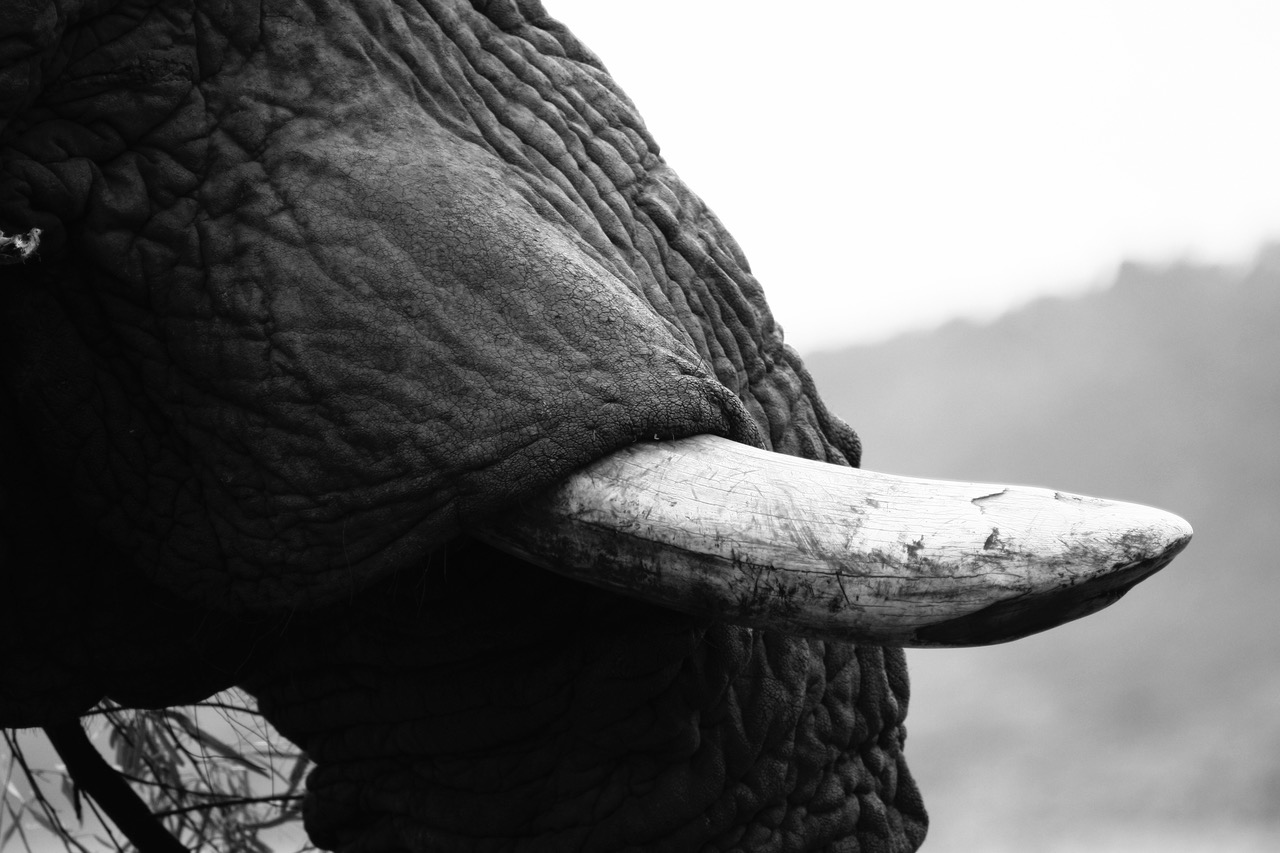

Supplement: Supplementary file 20 — Additional file 20. Elephant tusk. “Poaching to feed the elephant ivory trade is one of the most important threats to the conservation of this species. Elephants are one of the most charismatic animals of Africa and have been used as a flagship species for conservation efforts; conservation of elephants could mean conservation of African wild areas.” Attribution: Miguel Gomez (University of Manchester, UK). [file 12898_2017_138_MOESM20_ESM.jpg]

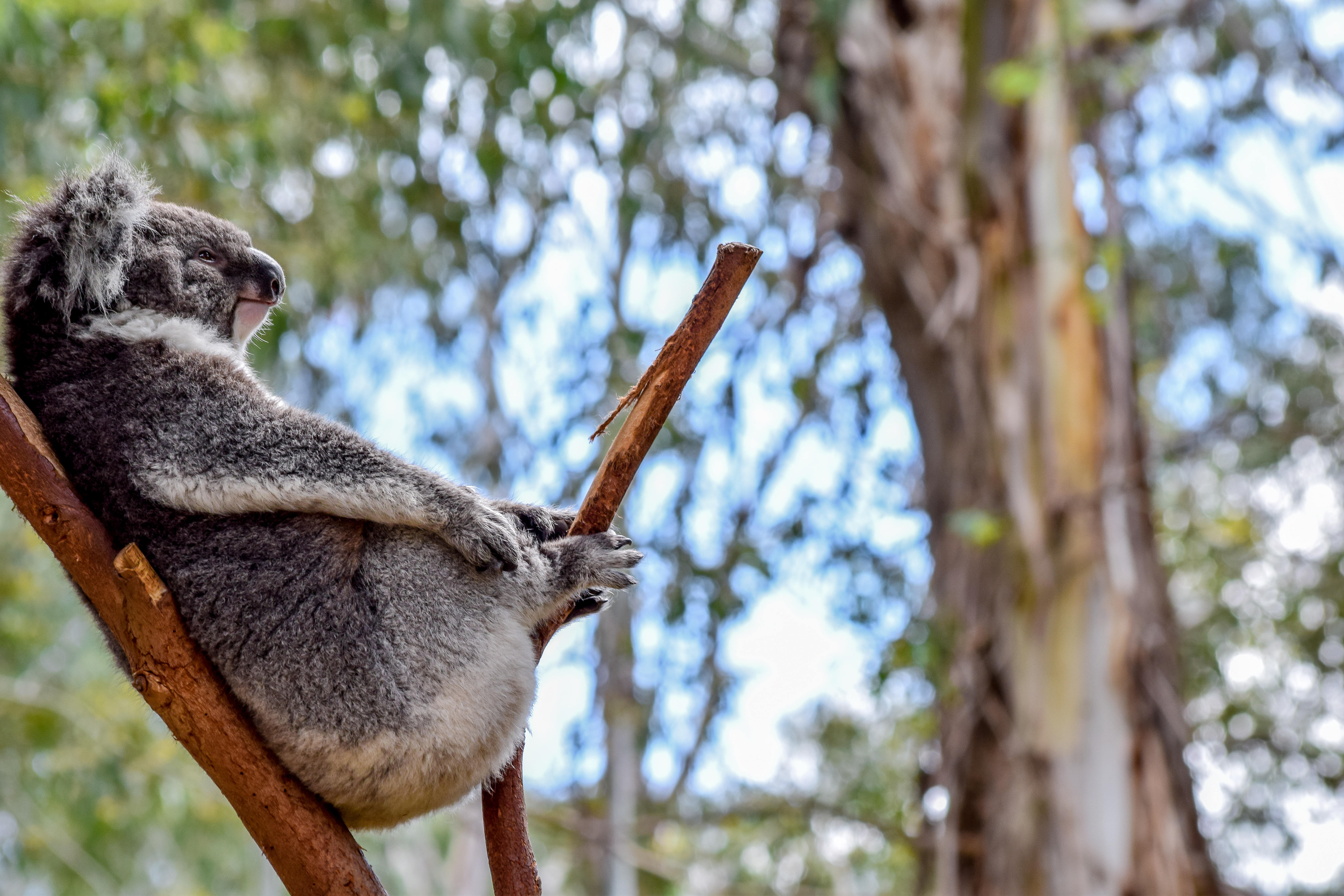

Supplement: Supplementary file 21 — Additional file 21. Peaceful koala. “This picture of a Koala sitting on a branch was taken around Canberra in Australia. Koalas are considered as vulnerable by the IUCN Red List of species and are subject to conservation programs in Australia.” Attribution: Arnaud Badiane (Macquarie University, Sydney, Australia). [file 12898_2017_138_MOESM21_ESM.jpg]

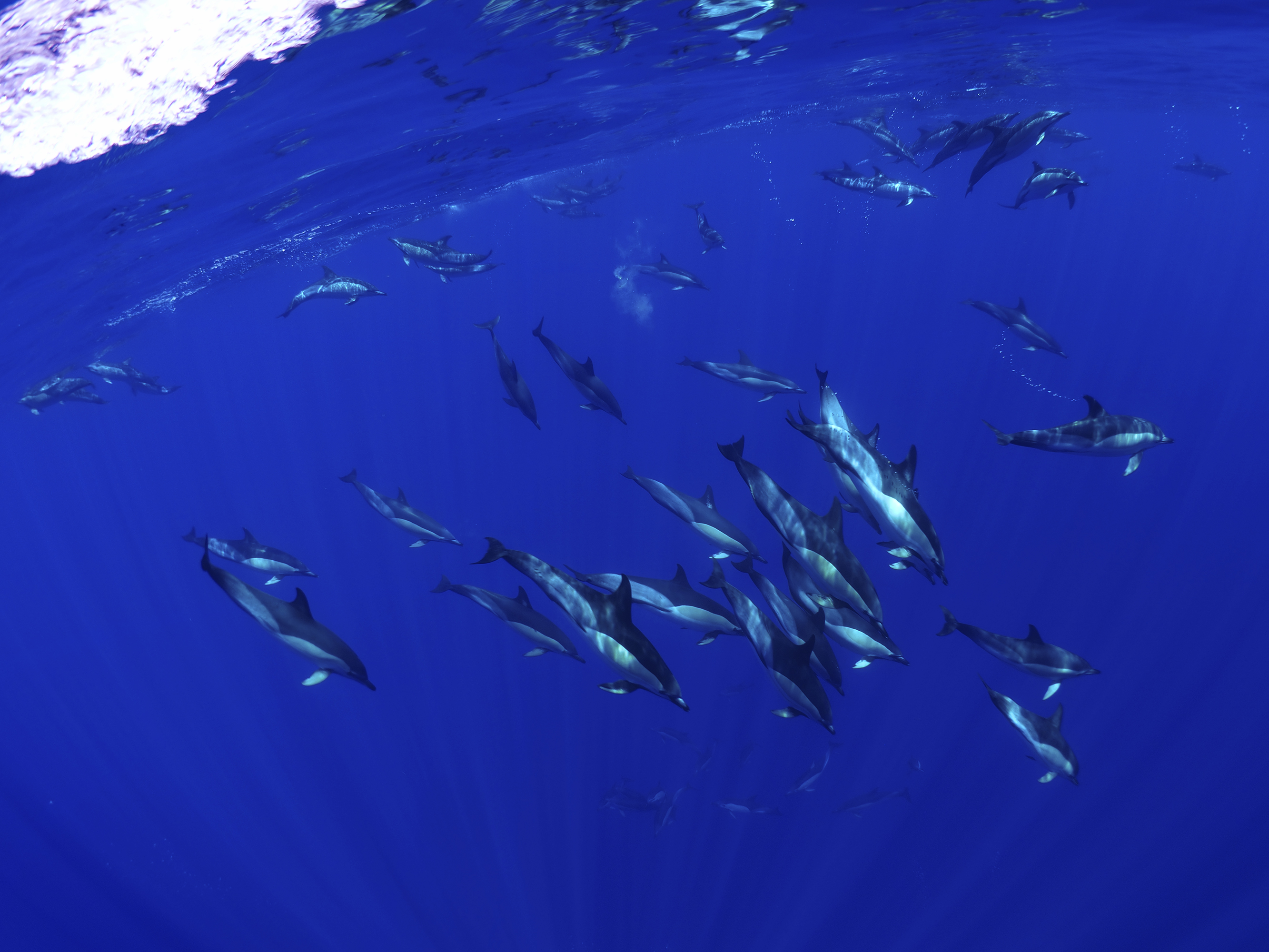

Supplement: Supplementary file 22 — Additional file 22. Dolphin school. “This photograph represents a part of a >100 individuals group of common dolphins (Delphinus delphis) in the oceanic waters of Madeira. It is difficult to describe the experience of being among these animals in their natural environment, everything is chaotic they swim around you, jump, the females approach you with their calves, it’s an overwhelming amazing experience.” Attribution: Diogo Sayanda (University of Lisbon, Portugal). [file 12898_2017_138_MOESM22_ESM.jpg]

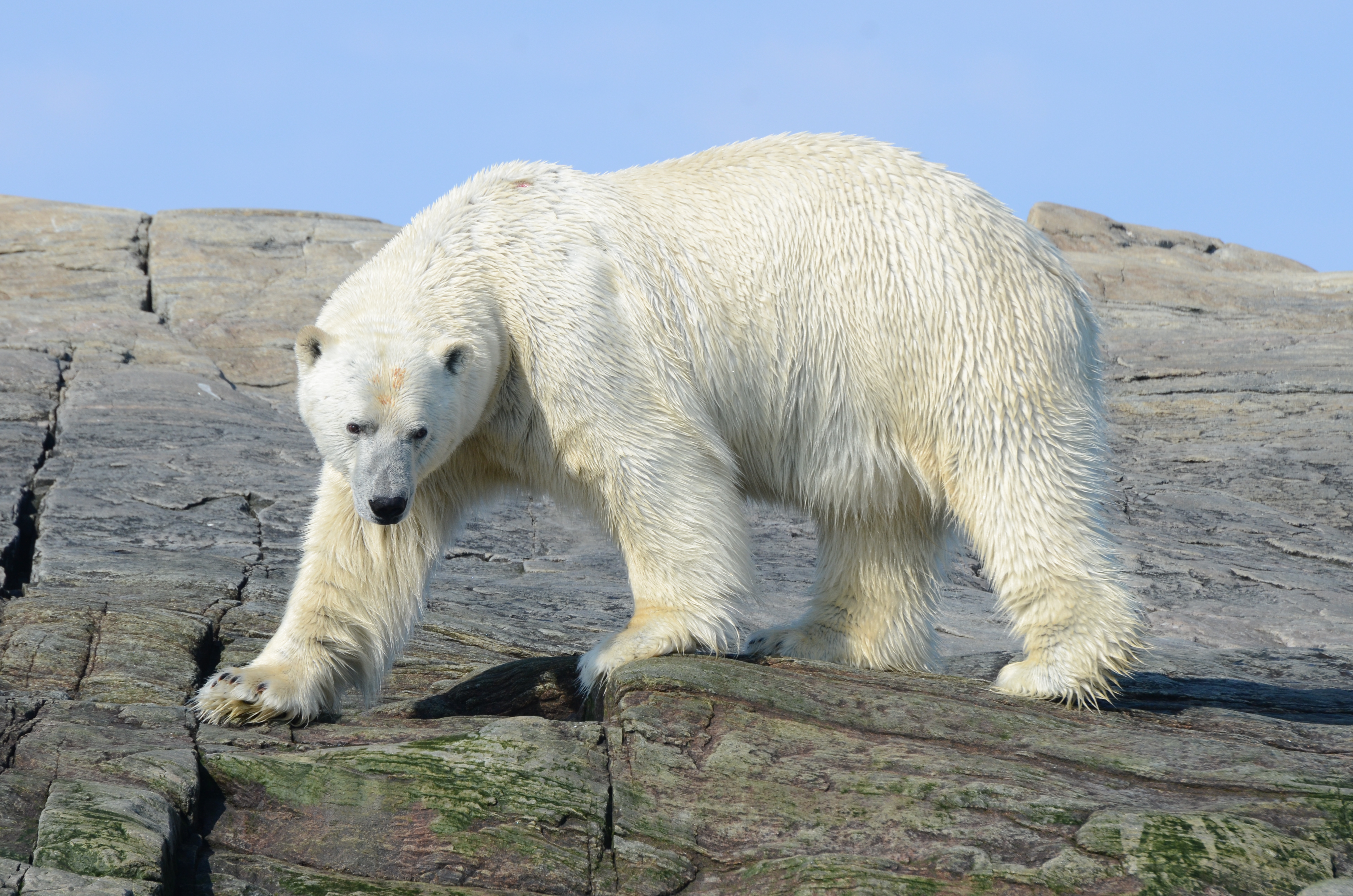

Supplement: Supplementary file 23 — Additional file 23. Polar bear. “Polar bear predation of common eider nests seems to be increasing because of sea ice loss. We are working to understand how sea ice loss is influencing polar bear foraging behaviour, and what effects this might have on common eider duck populations. This research is being done by boat-based surveys in the Hudson Strait, east of Cape Dorset in northern Canada, in close collaboration with local Inuit guides and assistants. On pre-surveyed islands, we counted the number of active and destroyed common eider nests, to quantify numbers of nest and their breeding success. We also recorded any polar bear signs, such as scat, footprints, and destroyed nests to quantify polar bear abundance. This image highlights the unpredictable nature of our work and sometimes you encounter a face to face with a giant male polar bear scavenging for eider eggs.” Attribution: Sjoerd Duijns (Carleton University, Canada). [file 12898_2017_138_MOESM23_ESM.jpg]

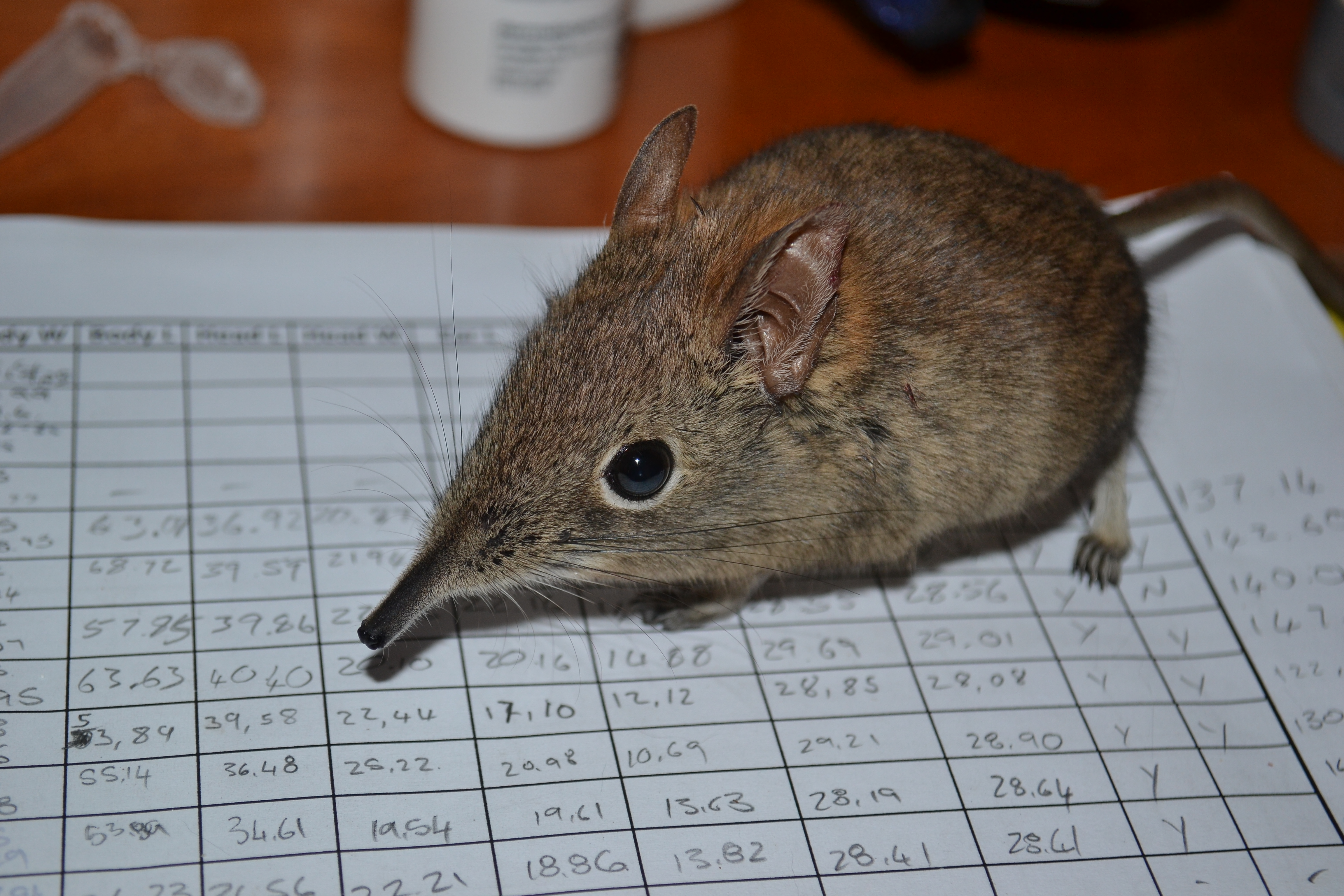

Supplement: Supplementary file 24 — Additional file 24. You better get the weight right! “I assisted with research on the behaviour of elephant shrews (Elephantulus myurus) trying to determine how personalities might affect their ecology. These animals have vastly different personalities, ranging from extremely aggressive to timid and some individuals are quite bold, as is the case for this individual, who is curiously exploring the research station, making sure its body measurements have been correctly recorded.” Attribution: Dewald Kleynhans (University of Pretoria, South Africa). [file 12898_2017_138_MOESM24_ESM.jpg]
